# Machine Learning‐Driven Design of Multicomponent Bone Inorganic Matrix Mimicking Scaffolds for Osteogenesis Enhanced by Neurogenesis

**DOI:** 10.1002/advs.77084

**Published:** 2026-08-27

**Authors:** Kunlu Lin, Ying Yang, Chuyao Zhou, Hanyue Mao, Zhiquan Zhang, Libangxi Liu, Xiaoying Yu, Haoming Liu, Long Liu, Xiaoyan Wang

**Affiliations:** ^1^ College of Science National University of Defense Technology Changsha Hunan People's Republic of China

**Keywords:** bone regeneration, high throughput screening, machine learning, mesoporous bioactive glass, neurogenesis

## Abstract

Bone defects require materials with osteogenic, neurogenic, and angiogenic activity, yet designing such materials within high‐dimensional compositional spaces remains challenging. Here, we report a machine learning (ML) driven strategy to accelerate the design of multicomponent mesoporous bioactive glasses (MBG). A dataset of 169 formulations was used to train a Support Vector Machine (SVM) model to classify osteogenic potential into two classes, high (relative ALP activity ≥0.83) and low (relative ALP activity <0.83). The model screened 10 000 candidates and identified 126 as high potential. These 126 candidates were then subjected to experimental mineralization assays using BMSCs, and selected four high‐performance candidates (Candidates 2–5) and one lower‐performance control (Candidate 1). The corresponding MBG cores (MBG1‐MBG5) were incorporated into NGF‐loaded silk fibroin shells to fabricate core–shell nanofibrous scaffolds via coaxial electrospinning. Among them, MBG5@SF/NGF exhibited the highest osteogenic activity, promoting BMSCs viability, differentiation, matrix mineralization, and Erk1/2 phosphorylation, along with upregulation of osteogenic and neuro‐inductive markers. Transcriptomic sequencing confirmed activation of osteogenic and neurogenic pathways. In mice calvarial defect model, MBG5@SF/NGF reconstructed a neuro‐vascular‐bone regenerative microenvironment. We conclude that the ML accelerated screening cascade, combined with experimental validation, identified MBG5@SF/NGF as a promising scaffold for functional bone regeneration.

## Graphical abstract

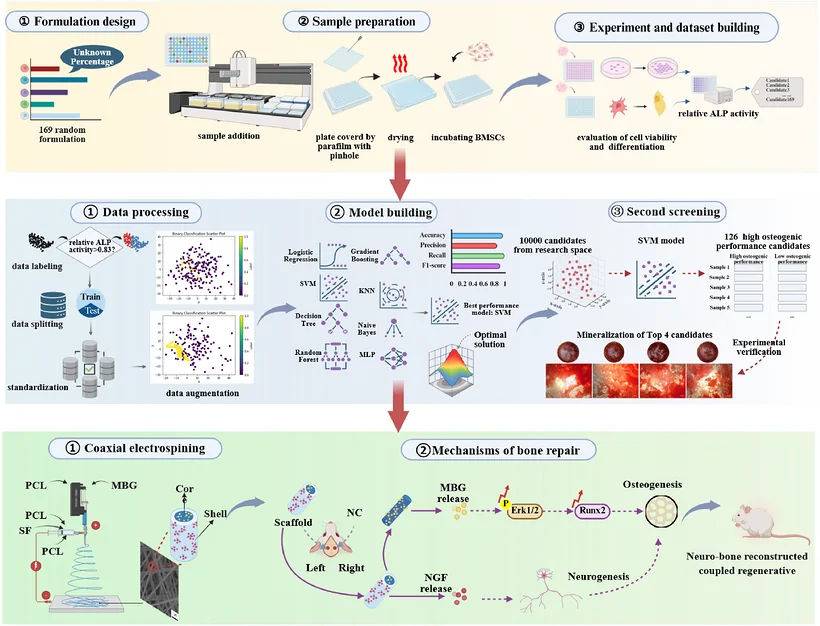

## 1 Introduction

Bone performs vital functions in the human body, including structural support, protection of internal organs, and enabling movement, as well as facilitating metabolism and hematopoiesis [1]. Bone defects caused by war injury, tumor resection, or congenital disorders present formidable clinical challenges [2]. This drives demand for advanced bone repair materials. These materials must not only restore structural integrity but also dynamically regulate the complex regenerative microenvironment [3]. An ideal bone graft should possess osteoconductivity, osteoinductivity, neurotrophic support, and angiogenic potential to achieve functional bone regeneration [4]. Among numerous biomaterials, mesoporous bioactive glass (MBG) has garnered significant attention due to its outstanding osteoconductivity, biocompatibility, and chemical composition similar to the bone mineral phase [5].

The inorganic phase of natural bone tissue constitutes approximately 70% of its dry weight [6]. Over 90% of this phase is hydroxyapatite, while the remaining approximately 10% comprises various key functional ions such as sodium, potassium, magnesium, and strontium [7]. Research indicates these trace elements play crucial regulatory roles in bone metabolism. Sodium and potassium ions help maintain cell membrane potential and osmotic pressure balance [8]. Magnesium ions serve as a cofactor for multienzymes involved in bone formation, and its deficiency is closely linked to osteoporosis [9]. Strontium ions have been demonstrated to bidirectionally regulate osteoclast and osteoblast activity, significantly enhancing bone strength [10]. Furthermore, citric acid may influence crystal nucleation, growth, and dissolution by chelating calcium ions on hydroxyapatite surfaces [11], while also participating in cellular energy metabolism. Thus, designing next‐generation biomimetic bone repair materials should not be limited to mimicking the primary apatite phase, but greater emphasis must be placed on the synergistic incorporation of these functional components to more precisely replicate the biochemical microenvironment of natural bone.

The design of bone‐mimetic inorganic matrix, such as MBG, involves multicomponents, forming a high‐dimensional compositional space. Traditional biomaterial development relies heavily on empirical trial‐and‐error approaches. Within the multicomponent, high dimensional compositional space, traditional approaches prove inefficient for design and struggle to systematically reveal synergistic effects between components, becoming a critical bottleneck in the development of high‐performance bone repair materials. In recent years, machine learning (ML) has brought a paradigm shift to materials science. By learning structure and property relationships from limited experimental data, ML can efficiently predict new material properties and reverse engineer composition combinations that meet multi‐objective requirements, significantly accelerating the material discovery process [12]. ML has been demonstrated potential for optimizing mechanical properties, bioactivity, and degradation behavior in polymer, alloy, and ceramic designs in the field of biomaterials [13]. However, no study has applied ML to the design of multicomponent MBG for osteogenesis coupled with neurogenesis.

**SCHEME 1:**
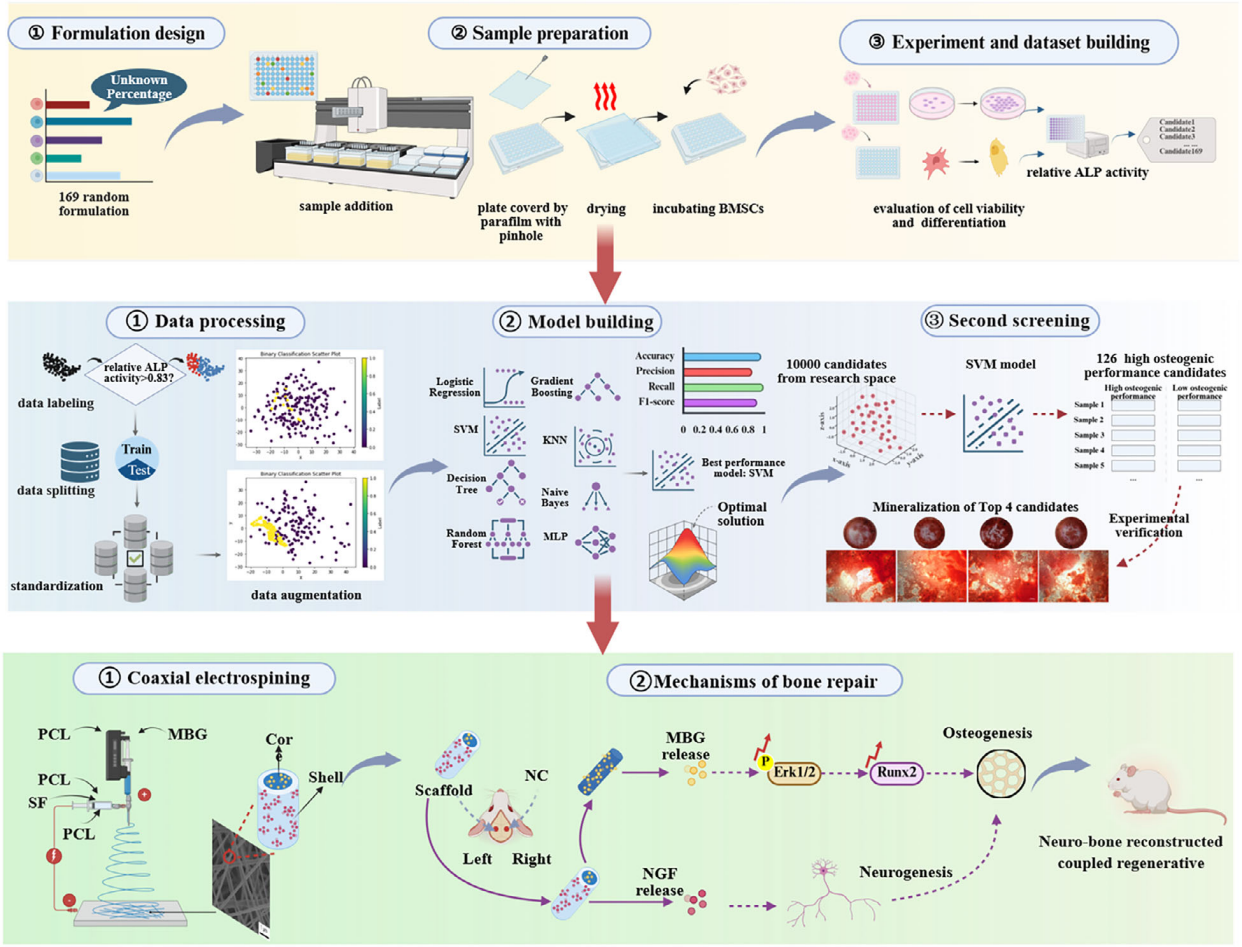
Schematic illustration of development of experimental datasets and algorithm models, screening and preparation of the scaffolds and their applications in vivo.

In this study, we construct an ML‐driven screening framework for multicomponent MBG, validate the osteogenic and neurogenic activities of the optimized MBG5@SF/NGF scaffold, and propose a neuro‐vascular‐bone coupled regenerative mechanism. We first constructed an experimental dataset comprising 169 distinct formulations with relative cell differentiation activity. Principal Component Analysis (PCA) and response surface modeling were employed to visualize regions of high‐activity formulations, while a Support Vector Machine (SVM) model was utilized for precise prediction of osteogenic potential. Using an automated high‐throughput experimental platform, we generated a dataset of 169 formulations, which was then used to train an SVM model. The model subsequently screened 10,000 in silico‐generated candidates and identified 126 with high predicted osteogenic potential were successfully identified. Subsequent experimental validation selected two optimal components as MBG3 and MBG5. MBG components as core and Nerve Growth Factor (NGF) adsorbed into silk fibroin (SF) as shell were fabricated into core–shell structured nanofiber scaffolds through coaxial electrospinning. We detected the osteogenic and neurogenic activity of these scaffolds. Meanwhile, we also determined the ability of bone defect repair accompanied with neuroregeneration and vascularization of these scaffolds in mice skull critical size defect model (Scheme 1). This study not only designed highly efficient bone repair materials but also established a generalizable ML‐assisted framework applicable to multicomponent biomaterial design. It provided a mechanistic explanation of how ion‐doped MBG synergistically regulates osteogenesis accompanied with neurogenesis and angiogenesis.

## 2 Results

### 2.1 Dataset and Model Performance

**FIGURE 1:**
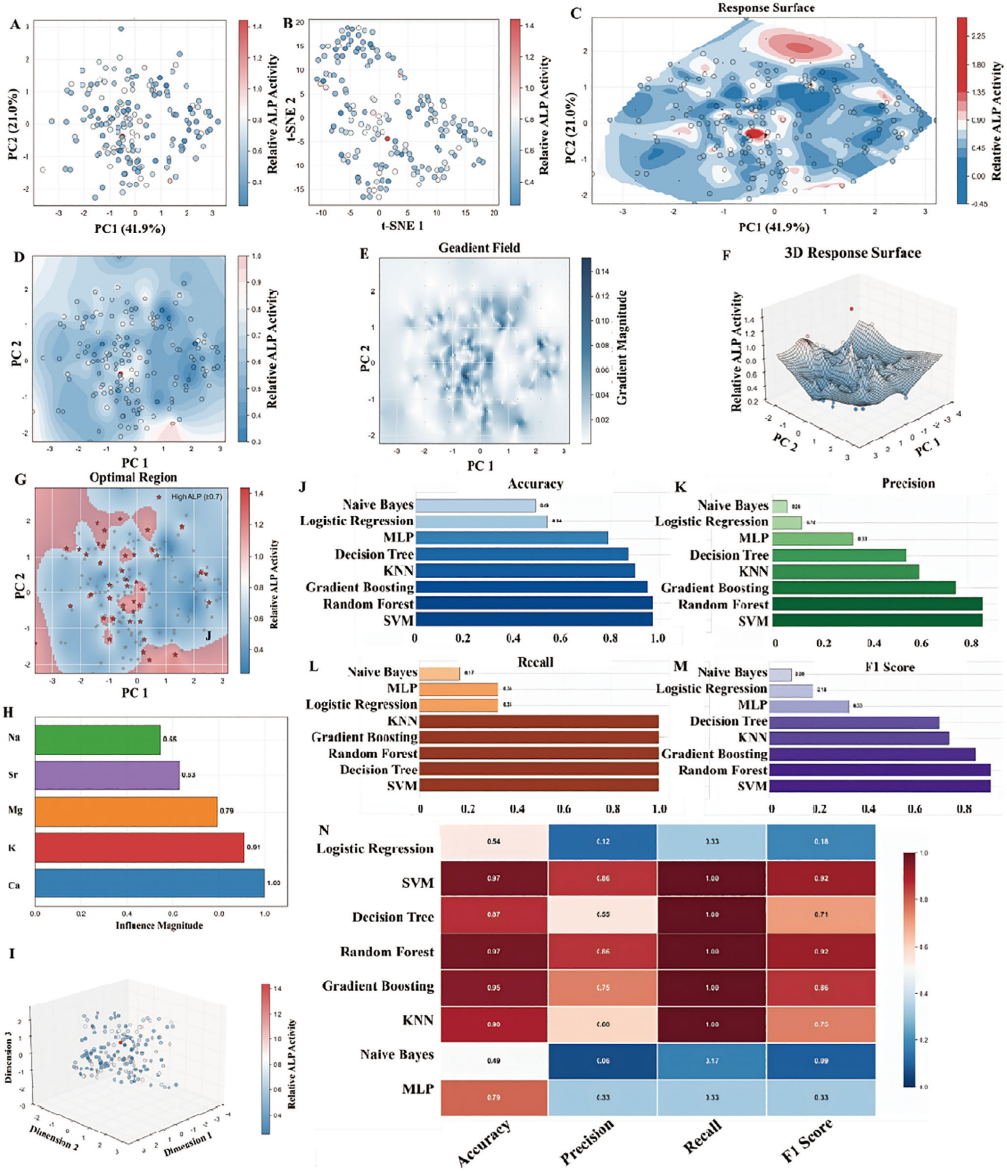
Dataset analysis and comparison of ML model performance. (A) PCA projection of the 169 formulation space, with colors indicating relative ALP activity. Red region represents high relative ALP activity while blue region represents low relative ALP activity. (B) Visualization using t‐SNE nonlinear dimensionality reduction. (C) Relative ALP activity response surface in the PCA reduced dimensional space. (D) Topographical features of relative ALP activity based on the PCA space. (E) Gradient field of the ALP topography, showing the direction of relative ALP activity change. (F) Three‐dimensional topographic map of relative ALP activity. (G) Definition of the high relative ALP activity region (threshold: 70th percentile). (H) Analysis of the elements component influence to algorithmic model. (I) Three‐dimensional PCA projection of each candidate. (J–N) Performance metric comparison of eight ML algorithms on the test set: accuracy (J), precision (K), recall (L), F1‐score (M), and a summary table of comprehensive performance metrics (N).

To establish the dataset, we first designed 169 distinct candidates with varying component ratios using a uniform design strategy, which efficiently covers the multi‐component composition space with a relatively small number of samples by maximizing spatial uniformity within the constrained total cation molarity framework. While 169 samples are modest for purely data‐driven ML, this scale is practically meaningful in biomaterial formulation screening and is substantially higher than the throughput achievable by conventional trial‐and‐error approaches. Preliminary materials corresponding to each candidate were synthesized via a dissolution‐drying method and subsequently subjected to assess BMSCs viability (CCK‐8 assay) and osteogenic induction (ALP activity assay). The result of CCK‐8, ALP activity, and relative ALP activity were shown in Tables S1–S3, respectively. The ALP/CCK‐8 ratio for each candidate was adopted as the evaluation metric for osteogenic performance as relative ALP activity. To visually explore the distribution of formulation compositions in relation to relative ALP activity, we employed Principal Component Analysis (PCA) as a visualization tool. The first two principal components (PC1 and PC2) cumulatively explained 41.9% and 21.0% of the variance, respectively indicating that this 2D projection effectively retained the structural information of the original data and can offers a useful overview, but it does not fully capture the complexity of the original 5‐dimensional space. Importantly, all subsequent SVM model training and predictions were performed in the complete 5‐dimensional original feature space. PCA served only for visualization and did not influence model construction or decision‐making (Figure 1A). The PCA projection map revealed a gradient distribution of relative ALP activity across the composition space. Samples with high relative ALP activity (red region) were predominantly concentrated in the area corresponding to PC1 = −0.5 and PC2 = −0.4 values. Actually, this spatial aggregation suggests the existence of specific formulation combinations that synergistically enhance relative ALP activity. To further validate the dimensionality reduction outcome, the t‐SNE nonlinear dimensionality reduction method was employed for comparison (Figure 1B). Although the specific axes differed, t‐SNE likewise exhibited a clustering tendency for relatively high ALP samples, which further corroborating the existence of an optimized composition subspace. The response surface of relative ALP activity within the PCA‐reduced dimensional space showed a distinct peak, with the maximum relative ALP activity occurring at approximately PC1 = −0.4 and PC2 = −0.3 (Figure 1C).

To gain deeper insight into the continuous variation of relative ALP activity within the composition space, we constructed a relative ALP response surface based on the PCA space (Figure 1D). By computing the gradient field of the response surface, we identified the direction of the steepest change in relative ALP activity (Figure 1E). From low relative ALP to high relative ALP regions formed a convergent and centripetal pattern, indicating the presence of optimization pathways attracting formulations toward higher relative ALP performance. Notably, within the region spanning PC1 from −0.8 to −0.2 and PC2 from −1 to −0.1, the gradient magnitude increased significantly, suggesting heightened sensitivity of relative ALP activity to component ratio changes in this area. In addition, the response surface exhibited characteristic peak‐valley terrain features, with the highest relative ALP region forming a prominent peak located near coordinates of approximately PC1 ≈ −0.5, PC2 ≈ −0.4 (Figure 1F). We defined the range from 70% to 100% of relative ALP activity as the High relative ALP region (Figure 1G), which encompassed approximately 40 samples. Component analysis of these high relative ALP samples revealed that their average Ca content (75.0%) was significantly higher than that of low relative ALP samples (70.0%, *p* <0.001), further confirming the positive promoting effect of Ca. Moreover, component influence analysis further quantified the contribution of each component to the composition space structure, which showed that Ca demonstrated the greatest influence (1.00), followed by K (0.91), Mg (0.79) and Sr (0.63), indicating their dominant roles in determining the structure (Figure 1H). Furthermore, a three‐dimensional PCA projection (Figure 1I) corroborated the findings from the two‐dimensional analysis. In the 3D space, high relative ALP samples formed a relatively compact cluster located in the quadrant with positive PC1, negative PC2, and positive PC3 directions. This spatial distribution pattern suggests that optimizing formulations may require simultaneous adjustment of component ratios across all the dimensions.

We compared the performance of 8 distinct ML algorithms (Logistic Regression, SVM, Decision Tree, Random Forest, Gradient Boosting, KNN, Naive Bayes, MLP) in predicting osteogenic performance of multicomponent biomaterial formulations. In terms of Accuracy, all algorithms except Logistic Regression and Naive Bayes performed well (Figure 1J). Regarding the Precision, SVM and Random Forest showed superior performance (Figure 1K). For the Recall, SVM, Decision Tree, Random Forest, Gradient Boosting, and KNN all achieved 100% (Figure 1L). As for the F1 Score, Random Forest and SVM still gained the best score (Figure 1M). According to the heatmap, which showed 8 algorithms and their corresponding performance, SVM and Random Forest demonstrate much better performance on our dataset (Figure 1N). Considering these metrics comprehensively, the SVM algorithm, which is more suitable for small sample [14], high‐dimensional datasets and demonstrated the best overall performance was selected.

### 2.2 Screening of Optimal Formulations

**FIGURE 2:**
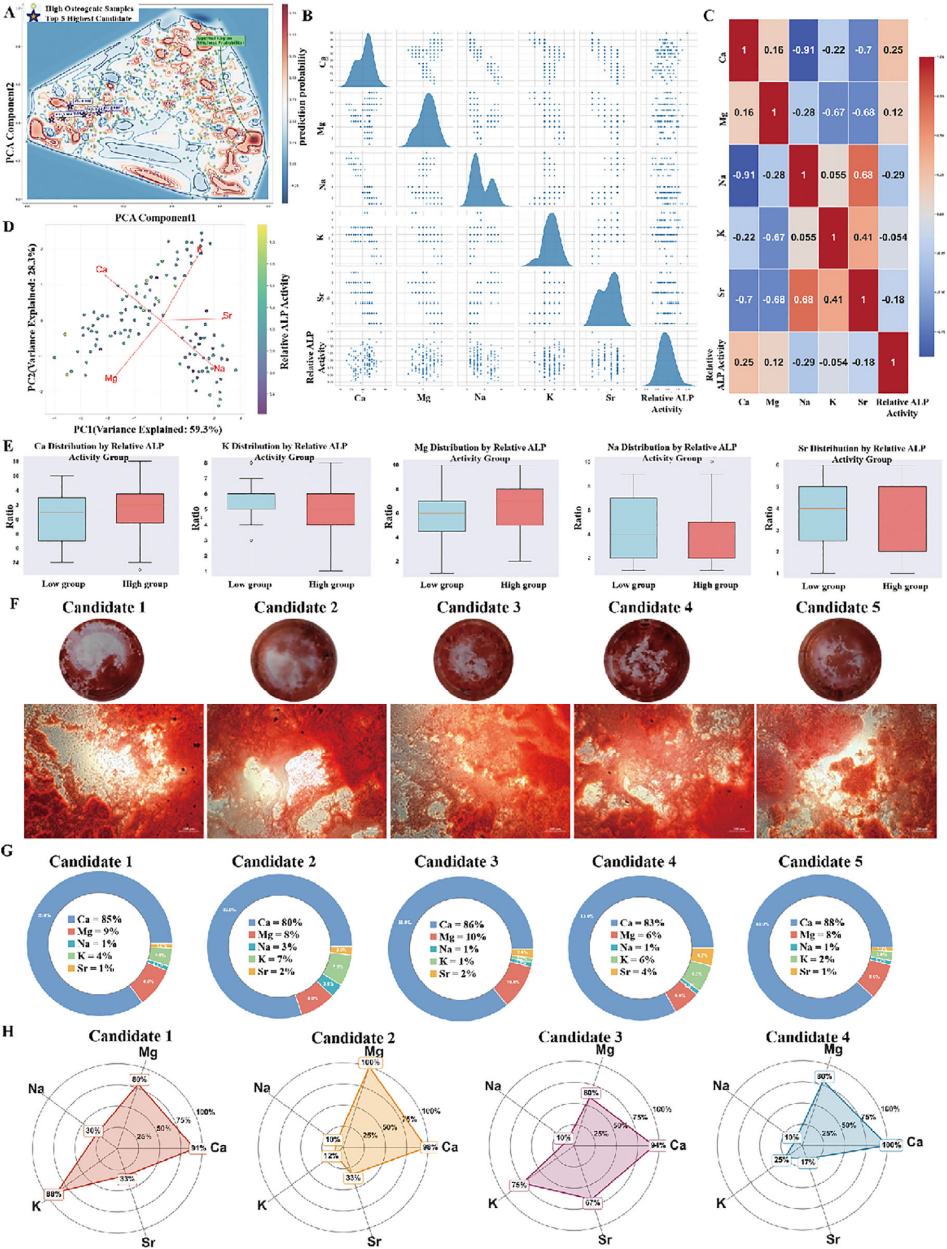
Optimal formulation screening and secondary validation. (A) Topographic map showing the distribution of formulations predicted as High Osteogenic Potential among 10 000 virtually generated formulations after PCA dimensionality reduction. Color intensity represents the prediction probability, and pentagrams mark the top 5 candidates with the highest prediction probabilities. (B) Relationship between the element components of the 126 candidate optimal formulations and the predicted relative ALP activity. (C) Heatmap displaying the interaction relationships among element components within these 126 formulations. (D) Scatter plot of the 126 candidate formulations after PCA dimensionality reduction. Arrows indicate the directions corresponding to different element components. (E) Box plots depicting the distribution of the five element component contents corresponding to high and low mineralized nodule deposition groups, divided by an absorbance threshold of 0.95. (F) Alizarin Red S staining showing the deposition of ECM mineralized nodules induced by different elements formulations in BMSCs. (G) Mole percentages of components for 1 low‐performance candidate and 4 high‐performance candidates. (H) Component ratio chart for the four high‐performance groups after second round screening.

Based on the SVM model, which exhibited optimal performance on the testing set, we predicted the osteogenic performance for 10 000 candidates generated via uniform design within the multicomponent search space. The predict result of 10 000 candidates were shown in Table S4. Among the 10 000 candidates, 126 (1.26%) were classified by the model as high osteogenic performance. To visually represent the relationship between 126 component ratios and osteogenic performance, a distribution topographic map was plotted after PCA dimensionality reduction (Figure 2A). Each point represents a candidate predicted to have high osteogenic performance, with red color intensity indicating the prediction probability. The distribution reveals clustered regions of high osteogenic potential. Five pointed stars mark the top 5 candidates with the highest predicted probabilities, which are concentrated near the center of the optimal region, representing the most promising candidates. The optimal region was identified, characterized by dark colored areas with prediction probabilities >0.9 and relatively gentle contour lines. This low percentage underscores the scarcity of the optimal region within the multidimensional composition space and highlights the necessity of ML‐guided targeted screening. Taken together, these results illustrate the relationships among the candidates and the predicted relative ALP activity for these 126 optimal candidates.

As shown in the Scatter Plot Matrix, we visualized the interaction relationships between the components and relative ALP activity of these 126 candidates (Figure 2B). On the other hand, the heatmap also displays the interaction relationships among components within these 126 candidates (Figure 2C). In addition, we used PCA for dimensionality reduction, and the multidimensional component ratios and mineralization performance were represented in a scatter plot (Figure 2D), where arrows indicate the directions of different candidates. Based on the quantitative analysis results, we set a threshold of 0.95 and generated box plots depicting the distribution of the components corresponding to high and low relative ALP activity values (Figure 2E). Furthermore, to conduct a second round of refined screening from these 126 candidates, we synthesized all candidate materials and quantitatively assessed their ability to induce cell mineralization via Alizarin Red S staining. The result of the mineralization experiment was shown in Table S5. We selected the 4 candidates with the highest mineralized nodule deposition as the high‐performance experimental groups (candidates 2–5), 1 candidate with the lower mineralized nodule deposition as the low‐performance experimental group (candidate 1) (Figure 2F). Meanwhile, the pie chart showed the mole percentages of components for the 5 candidates (Figure 2G). Finally, the radar chart showed the proportion of each component in their search space for the 5 candidates (Figure 2H). The component mass ratio of Ca:Mg:Na:K:Sr as follows: 85:9:1:4:1 (candidate1), 80:8:3:7:2 (candidate 2), 86:10:1:1:2 (candidate 3), 83:6:1:6:4 (candidate 4), 88:8:1:2:1 (candidate 5).

### 2.3 Algorithm Workflow

**FIGURE 3:**
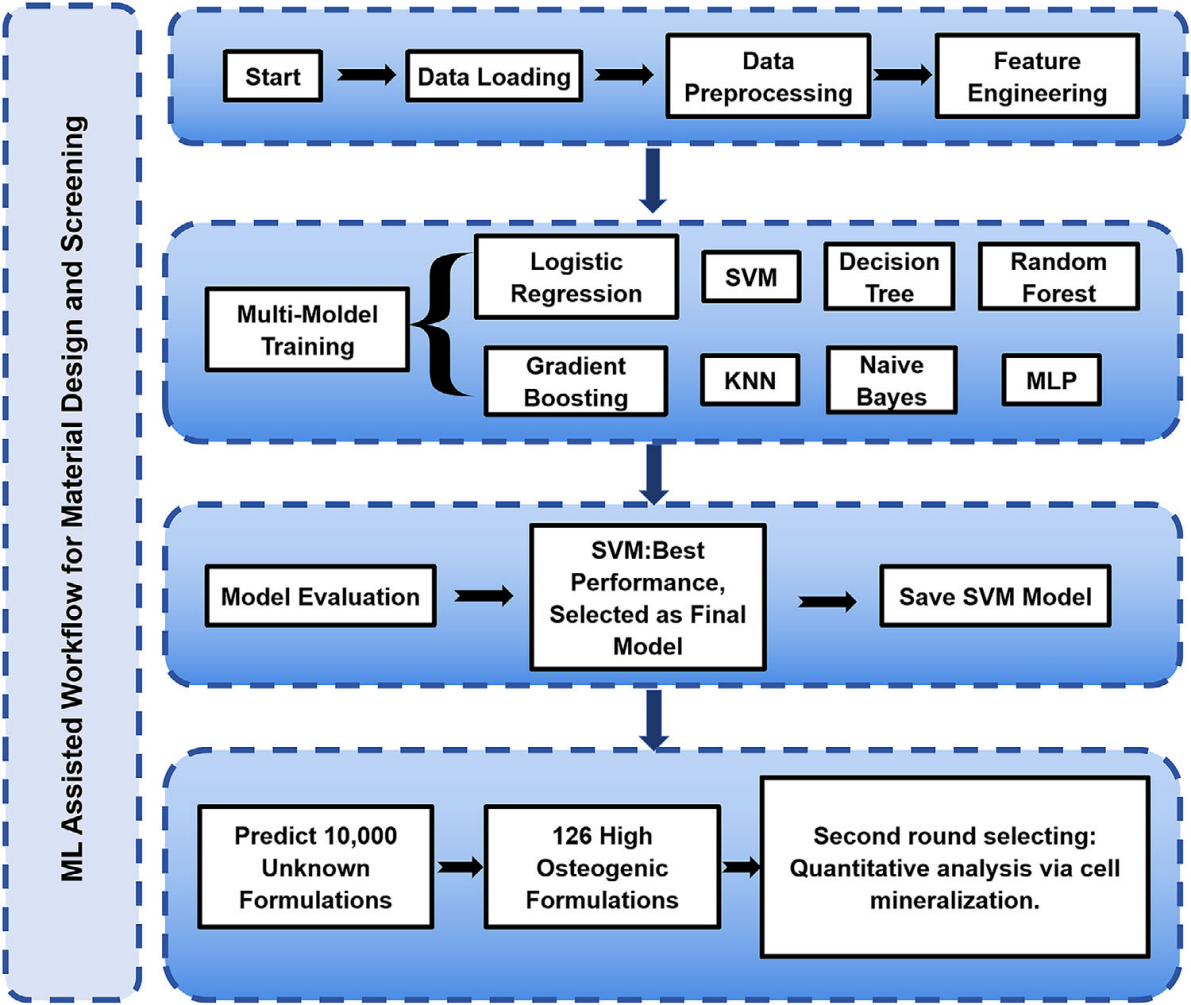
ML Assisted Workflow for Material Design and Screening.

The algorithm workflow integrates three sequential and interdependent stages, including data preprocessing and feature engineering, multimodel comparison and optimization, and final model deployment for predictive screening (Figure 3). In Data Preprocessing and Feature Engineering progress, the workflow commenced with data acquisition and label definition. When raw data were loaded, a binary classification label (high vs. low osteogenic potential) was assigned based on a relative ALP activity threshold of >0.83, primary compositional features were then extracted. To ensure a robust evaluation, the dataset was partitioned into training and testing subsets using a 7:3 ratio via stratified sampling, preserving the original class distribution. Addressing class imbalance, Synthetic Minority Over‐sampling Technique (SMOTE) was applied exclusively to the minority class samples within the training set during cross‐validation, while the test set remained untouched to prevent data leakage. Then, standardization was used to normalize feature scales and enhance model convergence. To identify the most robust predictive algorithm, eight distinct ML models were trained and evaluated in parallel, including Logistic Regression, SVM, Random Forest, Gradient Boosting, KNN, Decision Tree, Naïve Bayes, and MLP. For each model, hyperparameters were meticulously tuned using GridSearch CV coupled with fivefold stratified cross‐validation. The F1 score was adopted as the primary evaluation metric to optimize the balance between precision and recall. Following a comprehensive evaluation, the SVM model demonstrated superior predictive performance, attaining the highest F1 score, and was consequently selected as the final model for subsequent application. The last step is model application and high‐performance candidate screening. The optimized SVM model was deployed to screen a large library of 10,000 unexplored candidates. The new dataset underwent an identical preprocessing and feature engineering pipeline as the training data. The saved SVM model was loaded and utilized to compute osteogenic probability scores for each candidate. Subsequently, the SVM model selected 126 candidates with a high predicted probability of possessing superior osteogenic performance. A stringent confidence threshold (≥0.95) was applied to classify high‐potential candidates. Furthermore, we do the quantitative analysis via alizarin red staining after 14 days of mineralization induction of BMSCs as the second round selecting. This systematic approach successfully identified 4 candidates with high osteogenic performance, validating the efficacy of the established workflow for accelerated biomaterial discovery.

### 2.4 Material Characterization

**FIGURE 4:**
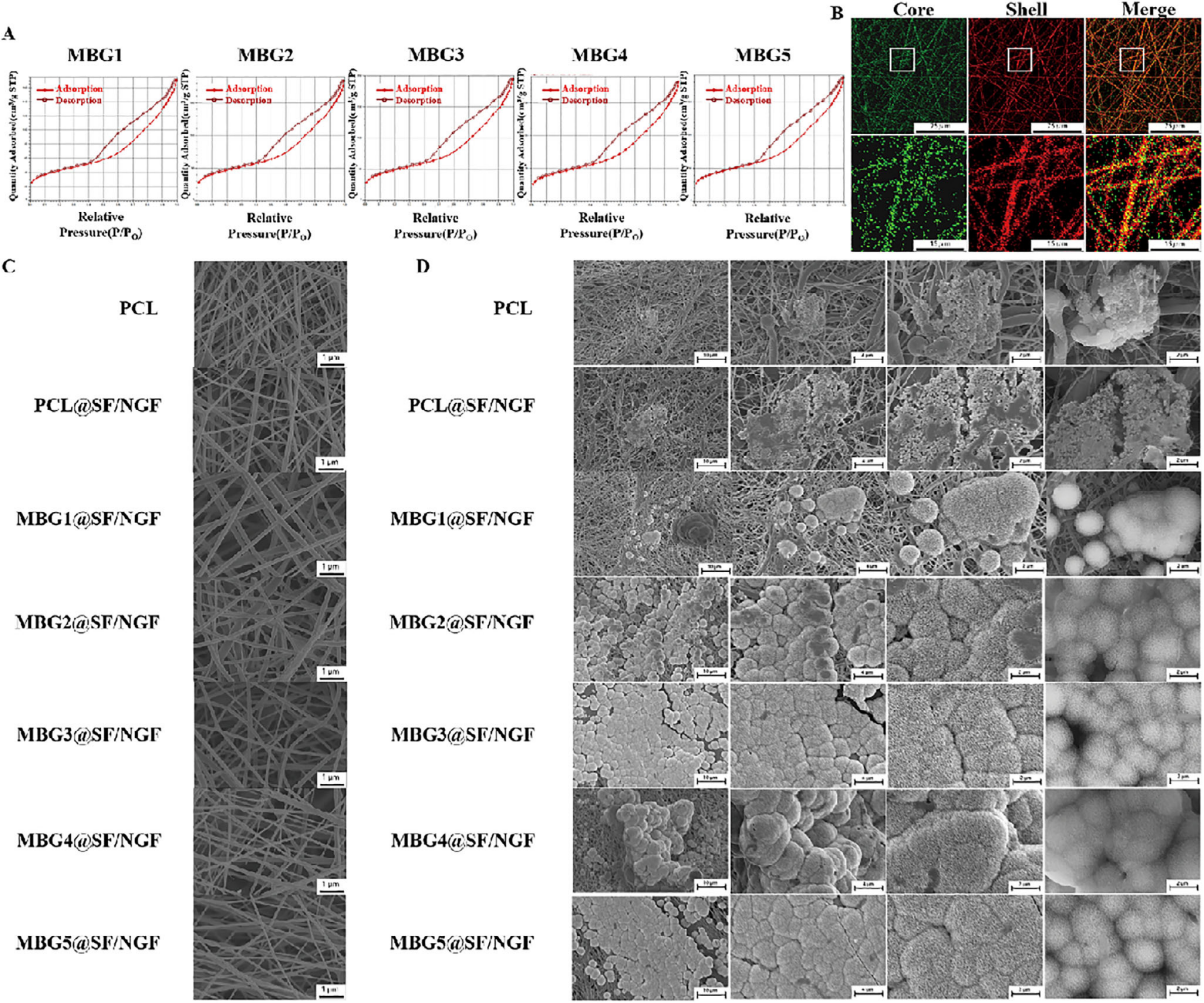
Materials and scaffolds characterization. (A) N_2_ adsorption–desorption isotherms of MBG1, MBG2, MBG3, MBG4, and MBG5. (B) Determination of the core–shell structure through CLSM images. (C) SEM detection of fibrous scaffolds. PCL, PCL@SF/NGF, MBG1@SF/NGF, MBG2@SF/NGF, MBG3@SF/NGF, MBG4@SF/NGF, and MBG5@SF/NGF. (D) SEM observation of mineralized deposition on the surfaces of PCL, PCL@SF/NGF, MBG1@SF/NGF, MBG2@SF/NGF, MBG3@SF/NGF, MBG4@SF/NGF scaffolds immersing in SBF for 14 days in vitro.

We characterized the screened component by ML was fabricated into MBG correspond to the component ratio as MBG1‐5. These BET results showed obvious hysteresis loops, which indicate that these MBG owning mesoporous were fabricated successfully (Figure 4A). All five ions exhibited sustained release from all MBG formulations over 9 days. Among the five formulations, MBG5 demonstrated the most stable release kinetics. We suggested that this steady and predictable ion supply may be particularly advantageous for bone regeneration, as it maintains effective concentrations of bioactive ions in the local microenvironment, thereby enabling sustained activation of osteogenic signaling pathways. MBG3 also exhibited relatively stable release behavior. In contrast, the remaining three formulations (MBG1, MBG2, and MBG4) showed noticeable fluctuations in their release profiles, with cumulative release curves displaying stepwise or alternating plateau and rise patterns, indicative of less uniform dissolution kinetics (Figure S1A–E). Furthermore, we fabricated the nanofibrous scaffold through coaxial electrospinning, with MBG in the core and SF/NGF in the shell. The core–shell structure was determined through Fluorescence staining with the core stained green by FITC and the shell stained red by Alexa Fluor 594, the merged image exhibited yellow, and the image below shows the zoom in figure of the area enclosed by the white box in the image above (Figure 4B). The surface morphology of these fibers showed the interwoven fibrous structures through SEM (Figure 4C). The mineralization effect of the high osteogenic performance groups was superior to that of the low osteogenic performance group (MBG1@SF/NGF) and the control group (PCL@SF/NGF), with MBG3@SF/NGF and MBG5@SF/NGF showing the best performance (Figure 4D). These results indicated that these good osteogenic performance fabricated scaffold own good mineralized ability in vitro.

### 2.5 Osteogenesis Determination

**FIGURE 5:**
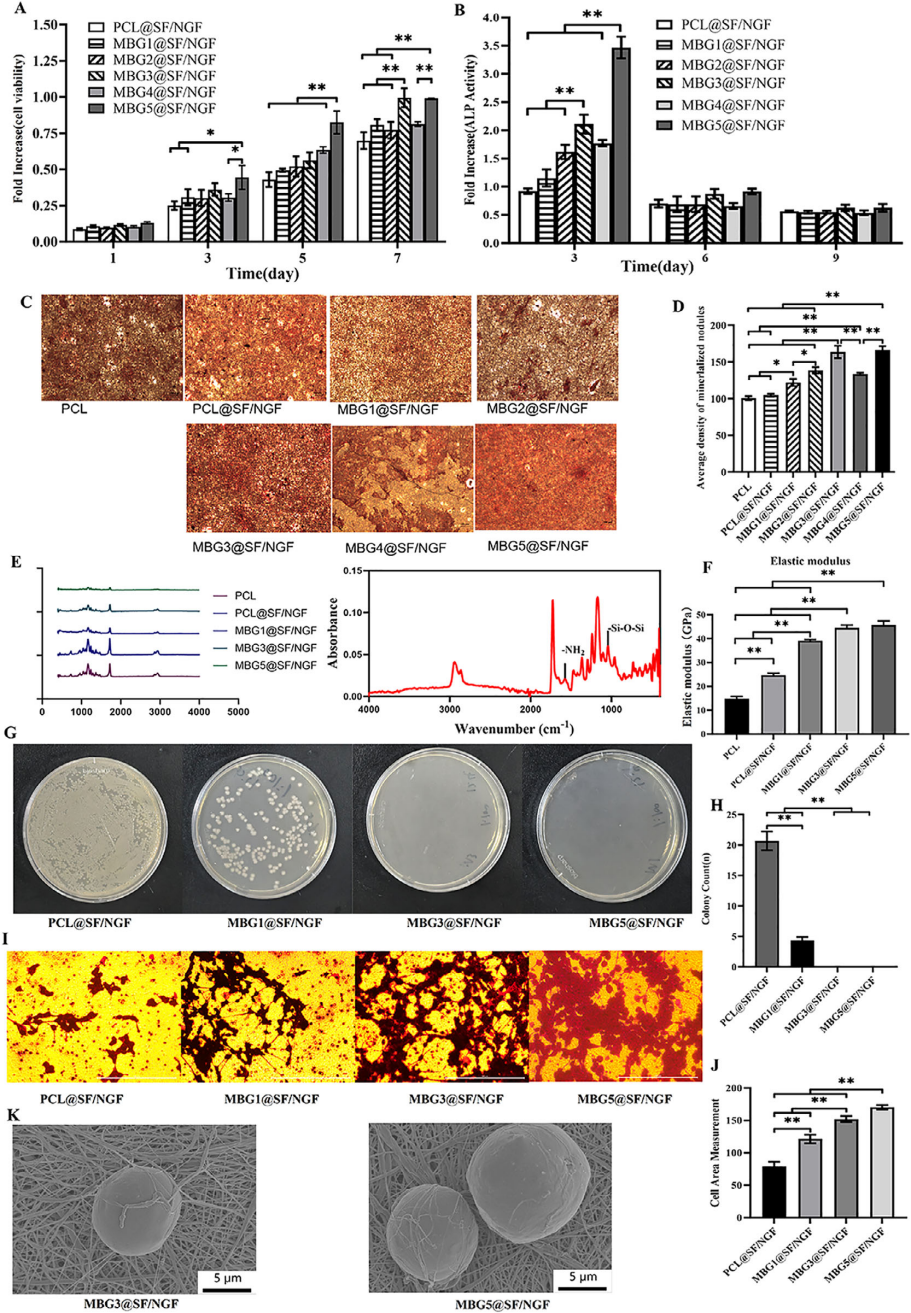
Osteogenic activity of these scaffolds. (A) Viability of BMSCs cultured on PCL@SF/NGF, MBG1@SF/NGF, MBG2@SF/NGF, MBG3@SF/NGF, MBG4@SF/NGF, and MBG5@SF/NGF scaffolds. (B) Differentiation of BMSCs cultured on PCL@SF/NGF, MBG1@SF/NGF, MBG2@SF/NGF, MBG3@SF/NGF, MBG4@SF/NGF, and MBG5@SF/NGF scaffolds. (C) Mineralization of BMSCs cultured on PCL, PCL@SF/NGF, MBG1@SF/NGF, MBG2@SF/NGF, MBG3@SF/NGF, MBG4@SF/NGF, and MBG5@SF/NGF scaffolds. Mineralized deposits were stained with Alizarin Red S. (D) Quantitative analysis of (C). (E) FTIR spectra of PCL, PCL@SF/NGF, MBG1@SF/NGF, MBG3@SF/NGF, and MBG5@SF/NGF scaffolds. (F) Tensile modulus of PCL, PCL@SF/NGF, MBG1@SF/NGF, MBG3@SF/NGF, and MBG5@SF/NGF scaffolds. (G) *E. coli* cultured on PCL@SF/NGF, MBG1@SF/NGF, MBG3@SF/NGF, and MBG5@SF/NGF scaffolds. (H) Quantitative analysis of (G). (I) Cell migration of BMSCs cultured on PCL@SF/NGF, MBG1@SF/NGF, MBG3@SF/NGF, and MBG5@SF/NGF scaffolds. (J) Quantitative analysis of (I). (K) SEM observation of cell adhesion of BMSCs cultured on MBG3@SF/NGF and MBG5@SF/NGF scaffolds.

To determine the effect of the scaffold to BMSCs, we detected the cell viability, differentiation, and mineralization. The result showed that the viability of BMSCs was higher cultured on the MBG5@SF/NGF scaffold compared that cultured on other scaffolds (Figure 5A). Moreover, the ALP activity of BMSCs cultured on the MBG5@SF/NGF scaffold was particularly significantly higher than that cultured on other scaffolds, exhibited highest on day 3 (Figure 5B), because ALP activity was the early marker of BMSCs differentiation and the expression level decreases over time. From the mineralization results, BMSCs cultured on MBG3@SF/NGF and MBG5@SF/NGF scaffolds formed a greater number of calcium nodules after 14 days (Figure 5C). Quantitative analysis revealed that the number of calcific nodules formed by MBG3@SF/NGF and MBG5@SF/NGF scaffolds was significantly higher than that in other groups. Furthermore, the number of calcific nodules in all groups supplemented with MBG materials exceeded that in the two PCL control groups (Figure 5D). The results of FTIR revealed that there is no significant different peaks between these scaffolds, and Si‐O‐Si bonds were formed in MBG, and a glass‐like structure was formed in the synthesized MBG, and the presence of ‐NH_2_ confirmed successful amino modification (Figure 5E). Furthermore, MBG3@SF/NGF and MBG5@SF/NGF scaffolds exhibited the best mechanical properties in tensile modulus analysis (Figure 5F). Specifically, the tensile modulus of MBG1@SF/NGF, MBG3@SF/NGF, and MBG5@SF/NGF is significantly higher than PCL@SF/NGF and PCL. This enhanced mechanical property is attributed to the uniform dispersion of MBG nanoparticles within the PCL core fibers, which serves as a reinforcing phase to improve the overall stiffness of the nanofibrous scaffold. Next, MBG3@SF/NGF and MBG5@SF/NGF scaffolds demonstrated the best antibacterial properties with almost no visible bacterial colonies, while the MBG1@SF/NGF group also showed significantly fewer colonies than the PCL@SF/NGF group, proving the excellent antibacterial capability of MBG‐doped materials (Figure 5G). Quantitative analysis of the antimicrobial experiment further corroborated the aforementioned results (Figure 5H). Besides that, significantly more BMSCs adhered to MBG5@SF/NGF scaffolds than that to other scaffolds, and more BMSCs adhered to MBG3@SF/NGF scaffold than that to PCL@SF/NGF and MBG1@SF/NGF scaffolds in cell migration detection (Figures 5I,J). Furthermore, the microscopic state of BMSCs in the scaffold was also examined. The result showed that BMSCs adherent to the inner part of MBG3@SF/NGF and MBG5@SF/NGF scaffolds (Figure 5K). Taken together, these results indicated that the ML‐screened MBG components, particularly MBG3 and MBG5, when fabricated into coaxial core–shell nanofibrous scaffolds, exhibit optimal osteogenic promotion, mineralization capacity, antibacterial activity, mechanical strength, and cell adhesion properties, making them as the promising candidates for bone tissue engineering applications.

### 2.6 Correlative Analysis of Signaling Pathways During Osteogenesis

**FIGURE 6:**
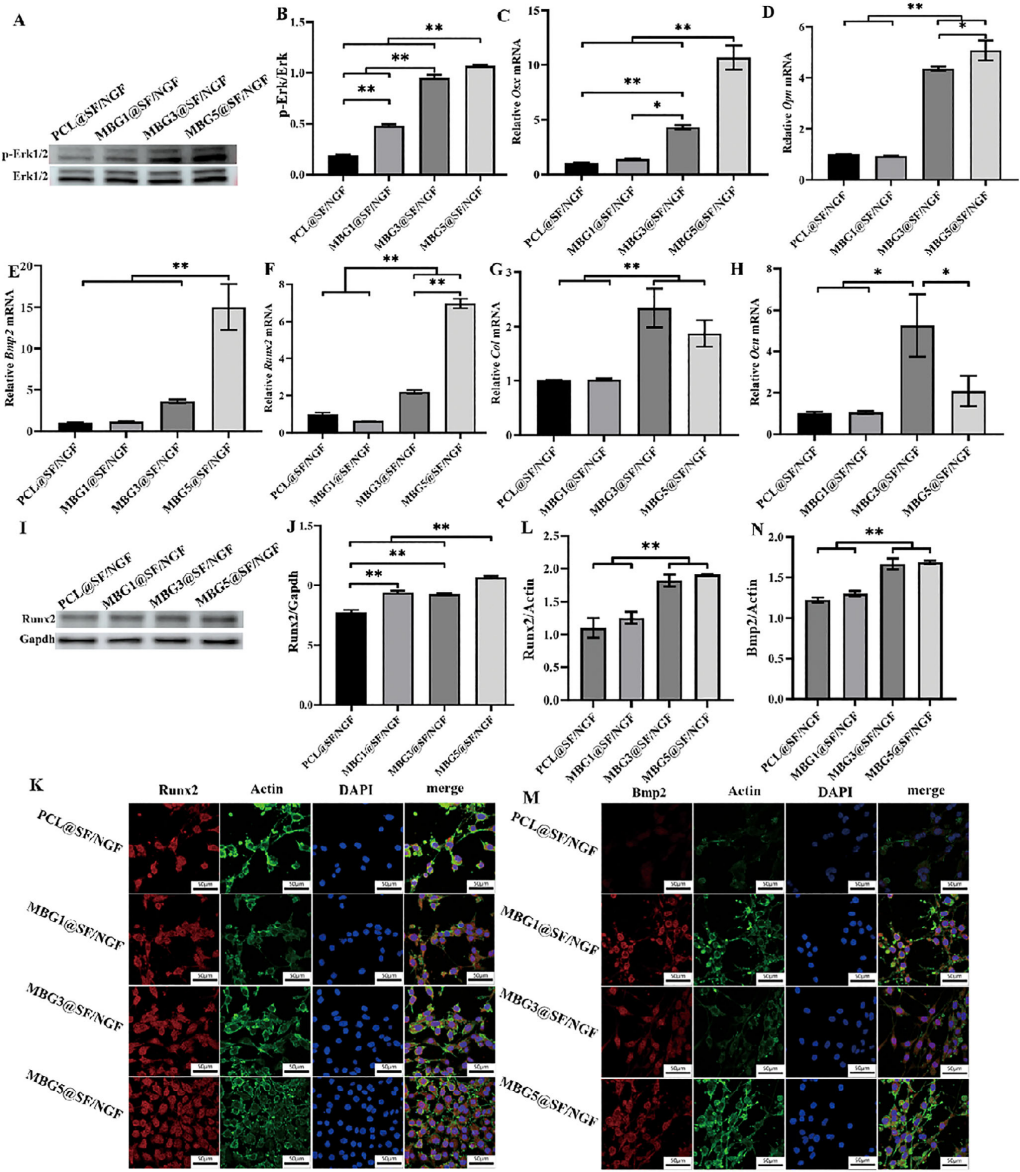
Osteogenic molecular mechanisms in BMSCs cultured on these scaffolds. (A) The phosphorylated Erk1/2 was detected, and the total protein levels of Erk1/2 was regarded as loading control. (B) Relative ratio of p‐Erk1/2 to Erk1/2 from the results in A. (C‐H) mRNA expression levels of osteogenic specific genes. The expression of *Osx* (C), *Opn* (D), *Bmp2* (E), *Runx2* (F), *Col* (G), and *Ocn* (H), with *Gapdh* as the internal control. (I) Runx2 protein expression was determined, and Gapdh protein level was regarded as loading control. (J) Relative ratio of Runx2 to Gapdh from the result in I. (K) Immunofluorescence of Runx2 (red). Co‐staining of Actin (green) and nuclei (blue). (L) Quantitative analysis of (K). (M) Immunofluorescence of Bmp2 (red). Co‐staining of Actin (green) and nuclei (blue). (N) Quantitative analysis of (M).

Erk1/2 signaling pathway plays a crucial role in the process of osteogenesis, enhancing cell viability, differentiation, and mineralization of osteoblasts. Phosphorylated Erk1/2 is its active form, and activated Erk1/2 is translocated into the nucleus to phosphorylate bone‐related transcription factors, thereby regulating the expression of osteogenic‐related genes [15, 16, 17]. Thus, the level of Erk1/2 phosphorylation in BMSCs cultured on these scaffolds was detected. The result showed that the phosphorylation level of Erk1/2 was higher in BMSCs cultured on MBG5@SF/NGF compared to that cultured on other scaffolds (Figure 6A,B). Moreover, we also determined the mRNA levels of osteoblastic‐specific marker genes. The result showed that mRNA levels of *Osx* (Figure 6C), *Opn* (Figure 6D), *Bmp2* (Figure 6E), *Runx2* (Figure 6F), and *Col* (Figure 6G) were higher in BMSCs cultured on MBG3@SF/NGF and MBG5@SF/NGF scaffolds compared with that cultured on other scaffolds, but the mRNA level of *Ocn* in BMSCs cultured on MBG3@SF/NGF scaffold was the highest than that on other scaffold (Figure 6H). We also determined the Runx2 for the protein level. The result showed that Runx2 level in BMSCs cultured on MBG5@SF/NGF scaffold was the highest compared with that cultured on other scaffolds (Figure 6I,J). Additionally, we also detected the cell expression level and location through immunofluorescence staining. The result showed that BMSCs cultured on these scaffolds exhibited well spread morphology, and Runx2 (red) displayed higher intensity fluorescent signals in BMSCs cultured on MBG3@SF/NGF and MBG5@SF/NGF scaffolds than that on other scaffolds, and the fluorescence signal was within the nucleus, colocalizing with the nucleus stained with DAPI (blue) which consistent with their effectively enhanced osteogenic differentiation activity (Figure 6K,L). The result showed that Bmp2 (red) also displayed higher intensity fluorescence in BMSCs cultured on MBG3@SF/NGF and MBG5@SF/NGF scaffolds compared with that on other scaffolds (Figure 6M,N). Taken together, these results suggested that MBG3@SF/NGF and MBG5@SF/NGF scaffolds promote osteogenic differentiation of BMSCs in conjunction with elevated p‐Erk1/2 levels and upregulation of Runx2 and downstream osteogenic genes.

### 2.7 Neurogenesis Determination

**FIGURE 7:**
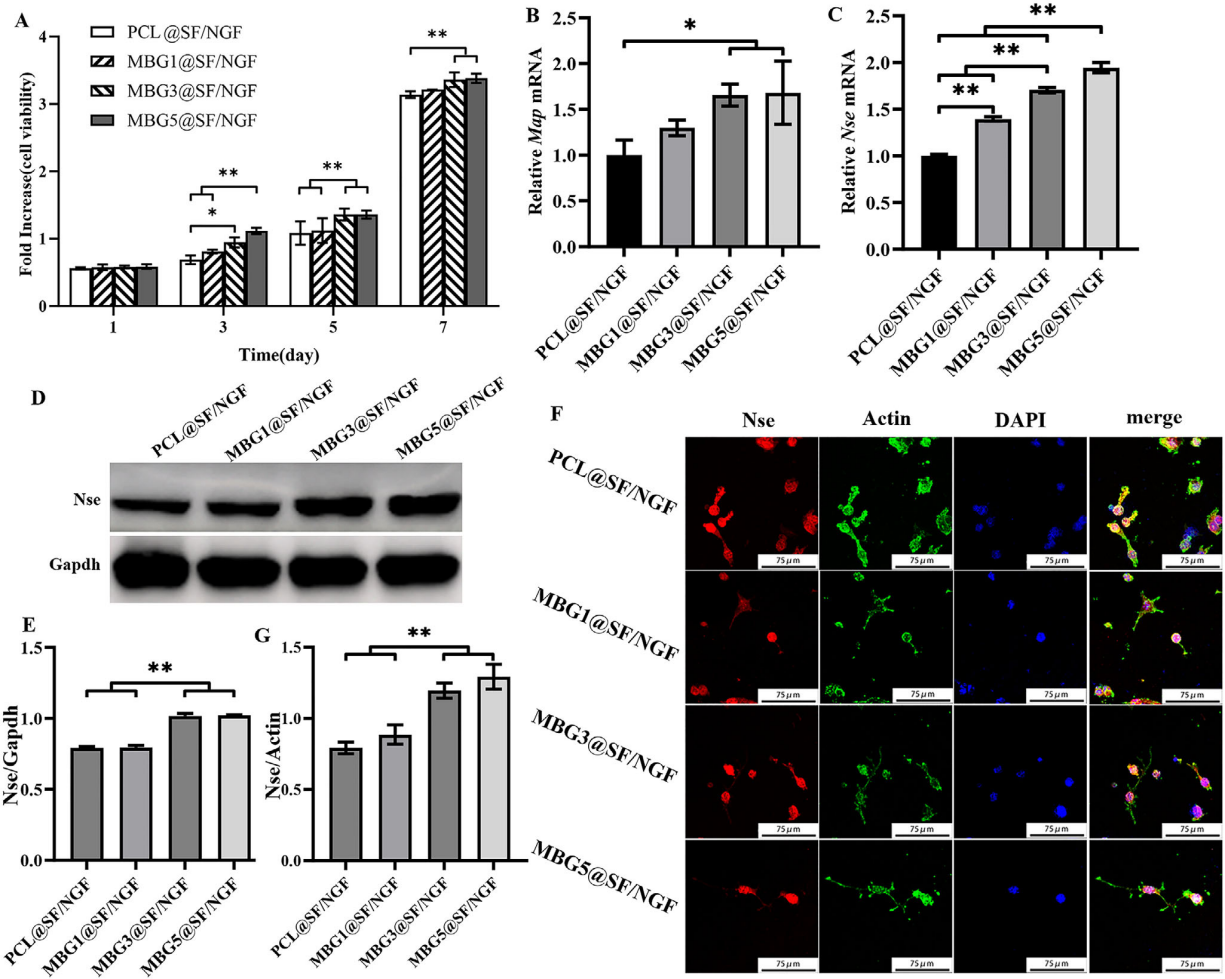
Neurogenic activity of these scaffolds. (A) Cell viability of BMSCs induced neural differentiation cells cultured on PCL@SF/NGF, MBG1@SF/NGF, MBG3@SF/NGF, and MBG5@SF/NGF scaffolds, respectively. (B‐C) mRNA expression levels of neurogenic specific genes, *Map* (B) and *Nse* (C), with Gapdh as the internal control. (D) Nse protein expression level with Gapdh as the loading control. (E) Quantitative analysis of (D). (F) Immunofluorescence of Nse (red) in BMSCs induced neural differentiation cells cultured on these scaffolds, respectively. Co‐staining of Actin (green) and nuclei (blue). (G) Quantitative analysis of (F).

The repair of nerves at the site of bone defects could enhance the process of bone repair. To investigate the effect of these scaffolds on neurogenesis, we determined the neural differentiation in BMSCs‐induced neural differentiation cells. The result showed that the viability of cells induced via BMSCs‐induced neural differentiation cells was significantly higher on MBG3@SF/NGF and MBG5@SF/NGF scaffolds than that cultured on PCL@SF/NGF scaffolds at day 3, 5, and 7 (Figure 7A). Moreover, the expression level of neuron‐specific marker gene was also increased in cells induced via BMSCs‐induced neural differentiation cells cultured on MBG3@SF/NGF and MBG5@SF/NGF scaffolds than that cultured on other scaffolds, *Map* (Figure 7B), and *Nse* (Figure 7C). Meanwhile, the protein expression level of Nse was also increased in cells induced via BMSCs‐induced neural differentiation cell cultured on MBG3@SF/NGF and MBG5@SF/NGF scaffold compared to that cultured on PCL@SF/NGF and MBG1@SF/NGF scaffold (Figure 7D,E). Furthermore, the cell morphology and the expression level of neuron‐specific marker protein were determined through immunofluorescence. The results showed that cells induced via BMSCs induced neural differentiation cell cultured on MBG3@SF/NGF and MBG5@SF/NGF scaffolds exhibited neuron‐like morphology, and the Nse protein expression level was also higher than that cultured on other scaffolds (Figure 7F,G). Thus, these results indicated that MBG3@SF/NGF and MBG5@SF/NGF scaffolds effectively possess neural inductive activity, as demonstrated by enhanced viability, elevated expression of neuron‐specific markers gene and proteins, and the development of neuron‐like morphology. The neural inductive capacity of these scaffolds may contribute to the creation of a pro‐regenerative microenvironment for bone repair, but definitive evidence of functional neurogenesis such as action potential firing, synaptic transmission, or neurotransmitter release awaits future electrophysiological investigation.

### 2.8 Transcriptomic Sequencing Reveals a Global Osteogenic Regulatory Network

**FIGURE 8:**
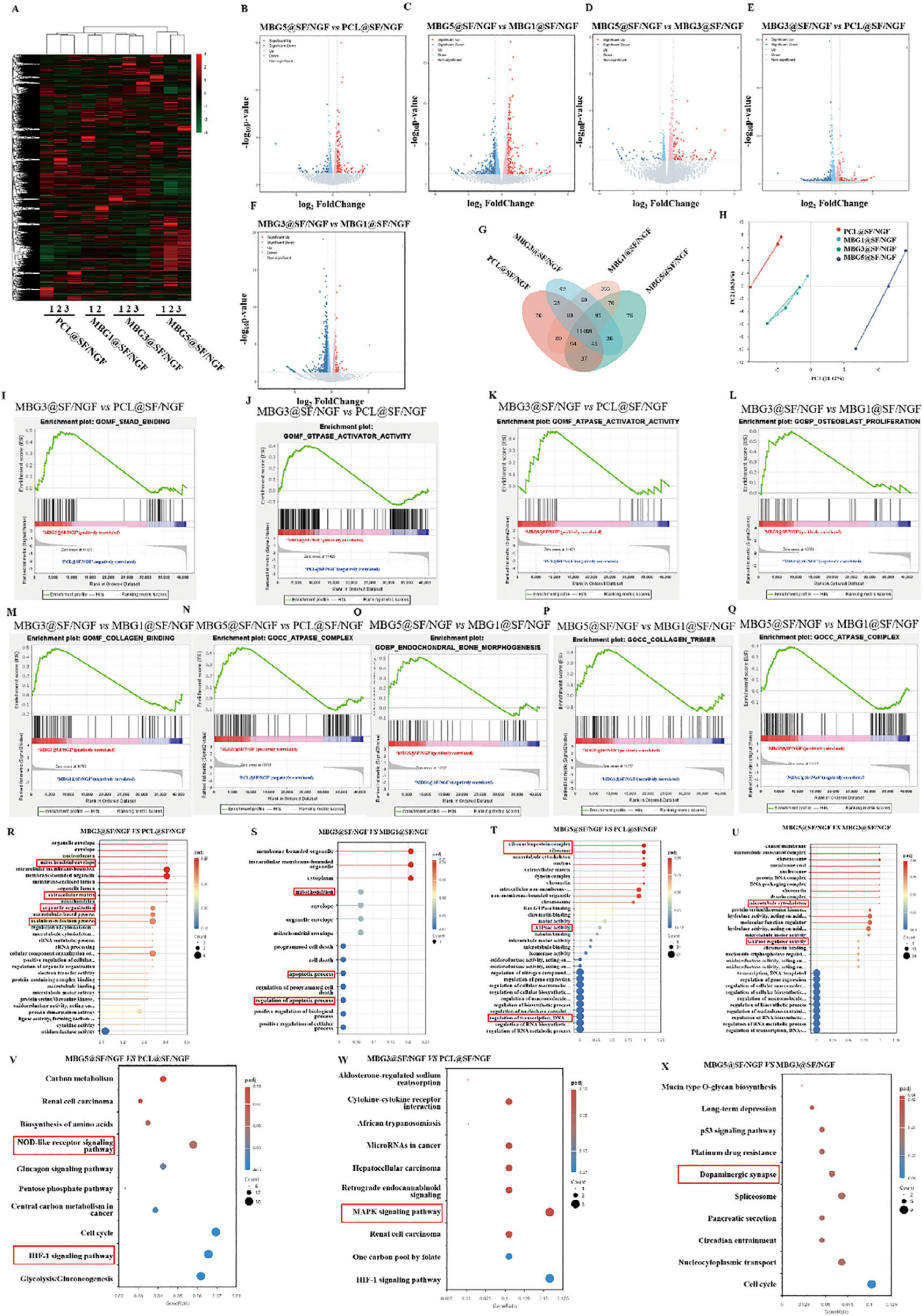
Transcriptome analysis during osteogenesis. Total RNA from BMSCs cultured on these scaffolds was used for transcriptome analysis. (A) The overall heatmap shows all differentially expressed genes. Hierarchical clustering analysis was used for the expression (FPKM values) of significantly expressed genes. (B‐F) Color‐coded volcano plots were drawn by counting all differentially expressed cells. MBG5@SF/NGF versus PCL@SF/NGF scaffold (B), MBG5@SF/NGF versus MBG1@SF/NGF scaffold (C), MBG5@SF/NGF versus MBG3@SF/NGF scaffold (D), MBG3@SF/NGF and PCL@SF/NGF scaffold (E), MBG3@SF/NGF and MBG1@SF/NGF scaffold (F). Red and blue coded points were represented gene sets with increased and decreased expression, respectively. (G) The gene convergence was showed through Venn diagram. (H) Sample clustering plot after dimensionality reduction. (I–Q) GSEA individual pathway analysis. Enrichment of SMAD (I), GTPASE (J) and ATPASE (K) in MBG3@SF/NGF versus PCL@SF/NGF scaffold. Enrichment of OSTEOBLAST (L) and COLLAGEN (M) in MBG3@SF/NGF versus MBG1@SF/NGF scaffold. Enrichment of ATPASE (N) in MBG5@SF/NGF versus PCL@SF/NGF scaffold. Enrichment of ENDOCHONDRAL_BONE_MORPHOGENESIS (O), COLLAGEN (P) and ATPASE (Q), in MBG5@SF/NGF versus MBG1@SF/NGF scaffold. (R–U) GO functional enrichment analysis results. MBG3@SF/NGF versus PCL@SF/NGF (R), MBG3@SF/NGF versus MBG1@SF/NGF (S), MBG5@SF/NGF versus PCL@SF/NGF (T), MBG5@SF/NGF versus MBG3@SF/NGF (U). (V–X) KEGG pathway enrichment analysis results. MBG5@SF/NGF versus PCL@SF/NGF (V), MBG3@SF/NGF versus PCL@SF/NGF (W), MBG5@SF/NGF versus MBG3@SF/NGF (X).

To further explore the effect of osteogenesis in BMSCs cultured on these scaffolds, we determined the mRNA level changes by RNA‐seq, and the expression profiles were also determined. Through Heatmap and volcano plot analysis, the results showed that gene differential expression in BMSCs cultured on different scaffolds. Compared to the PCL@SF/NGF scaffold, the MBG5@SF/NGF scaffold showed a differentially expressed gene percentage of 0.86% (Figure 8A,B). Compared to the MBG1@SF/NGF scaffold, the MBG5@SF/NGF scaffold had 2.28% (Figure 8A,C). Compared to the MBG3@SF/NGF scaffold, MBG5@SF/NGF scaffold accounted for 0.61% (Figure 8A,D). Meanwhile, the MBG3@SF/NGF scaffold versus the PCL@SF/NGF scaffold exhibited 0.94% (Figure 8A,E), and MBG3@SF/NGF scaffold versus MBG1@SF/NGF scaffold showed 2.29% differentially expressed genes (Figure 8A,F). These findings indicated that the highly osteogenic MBG3@SF/NGF and MBG5@SF/NGF scaffolds could influence mRNA expression, which participates in the osteogenic process. There were 49 and 76 genes exhibit differential expression in the MBG3@SF/NGF and MBG5@SF/NGF group respectively, which were further analyzed in subsequently conducted (Figure 8G). Clustering results after dimensionality reduction showed distinct groups with significant differences between different scaffold groups (Figure 8H).

To further analyze the molecular mechanisms, we performed Gene Set Enrichment Analysis (GSEA) (Figure 8I–Q), GO analysis (Figure 8R–U), and KEGG analysis (Figure 8V–X). We found that GSEA analysis revealed activation of a key signaling hub at the molecular function level: significant enrichment of SMAD BINDING‐related gene sets in the MBG3@SF/NGF group versus the PCL@SF/NGF group (Figure 8I). Smad proteins serve as direct downstream transcription regulators of TGF‐β/Bmp family signaling. This finding indicates that the material's pro‐osteogenic effect likely arises through activation of the classical Bmp‐Smad signaling axis. Comparisons between MBG3@SF/NGF versus PCL@SF/NGF, as well as MBG5@SF/NGF versus PCL@SF/NGF, consistently demonstrated enhanced cellular metabolic capacity promoted by the materials (Figure 8J,K,N). Furthermore, the MBG3@SF/NGF group exhibited enrichment in OSTEOBLAST PROLIFERATION (Figure 8L) and COLLAGEN BINDING (Figure 8M) compared to the MBG1@SF/NGF group. OSTEOBLAST PROLIFERATION directly confirms the osteogenic specificity of the scaffold. The enrichment of COLLAGEN BINDING suggests increased expression of cell surface or secreted Collagen‐binding proteins. We found that MBG5@SF/NGF exhibited enrichment in ENDOCHONDRAL BONE MORPHOGENESIS (Figure 8O), COLLAGEN_TRIMER (Figure 8P), and ATPASE COMPLEX (Figure 8Q) compared to MBG1@SF/NGF. The enrichment of ENDOCHONDRAL BONE MORPHOGENESIS indicates that the scaffold successfully activated and reproduced the core bone development and repair program in vivo. The enrichment of COLLAGEN_TRIMER suggests that under the influence of the scaffold, osteoblasts (or precursor cells) extensively synthesized and correctly assembled Collagen. This signifies that bone repair has entered the substantive matrix deposition stage. Meanwhile, ATPASE COMPLEX reveals the energy mechanism underlying bone repair.

GO enrichment analysis of the MBG3@SF/NGF versus PCL@SF/NGF group showed that under the influence of osteogenic‐inducing scaffolds, upregulating genes related to energy metabolism, organelle remodeling, and ECM construction, indicating that cellular function has shifted from proliferation to osteogenic differentiation and matrix secretion (Figure 8R). As for the MBG3@SF/NGF versus MBG1@SF/NGF group, the enrichment of mitochondrion, apoptotic process, regulation of the apoptotic process indirectly support osteogenic differentiation (Figure 8S). Meanwhile, analysis of MBG5@SF/NGF versus PCL@SF/NGF showed that BMSCs enriched genes related to ribosome structure, ATPase activity, and transcriptional regulation of DNA, providing the structural and functional foundation for subsequent osteogenic differentiation and matrix secretion (Figure 8T). Moreover, analysis of MBG5@SF/NGF versus MBG3@SF/NGF showed that the most striking changes in BMSCs were concentrated in components accompanied by enrichment in microtubule cytoskeleton and GTPase regulator activity (Figure 8U).

KEGG pathway enrichment analysis of differentially expressed genes revealed that when MBG5@SF/NGF compared with PCL@SF/NGF (Figure 8V). MBG5@SF/NGF significantly enriched the NOD‐like receptor signaling pathway, initiating an immune response conducive to repair. Concurrently, the HIF‐1 signaling pathway, acting as a core regulatory hub, was induced and activated. This pathway simultaneously drives angiogenesis to restore blood supply to the defect site and guides cellular metabolic reprogramming. The result revealed a marked enrichment of the MAPK signaling pathway in MBG3@SF/NGF versus PCL@SF/NGF (Figure 8W). This pathway plays a crucial role in regulating the proliferation and osteogenic differentiation of BMSCs, which is consistent with the results in Figure 6A,B. Comparing MBG5@SF/NGF with MBG3@SF/NGF, we observed significant activation of the Dopaminergic synapse pathway (Figure 8X). This provides a molecular‐level explanation for the neurogenic function of the scaffold, demonstrating its ability to enhance neurotransmitter‐related signaling. Taken together, these results suggested that MBG3@SF/NGF and MBG5@SF/NGF group promoted the osteogenic differentiation, Collagen synthesis, and energy metabolism of BMSCs by activating key signaling pathways such as BMP‐SMAD, MAPK, and HIF‐1, while also upregulating genes related to the cell cycle and immune regulation. In addition, the MBG5@SF/NGF enriched pathways associated with osteogenesis and neurogenesis, suggesting its potential for neuroregulatory effects while inducing bone regeneration.

### 2.9 Osteogenesis in Vivo

**FIGURE 9:**
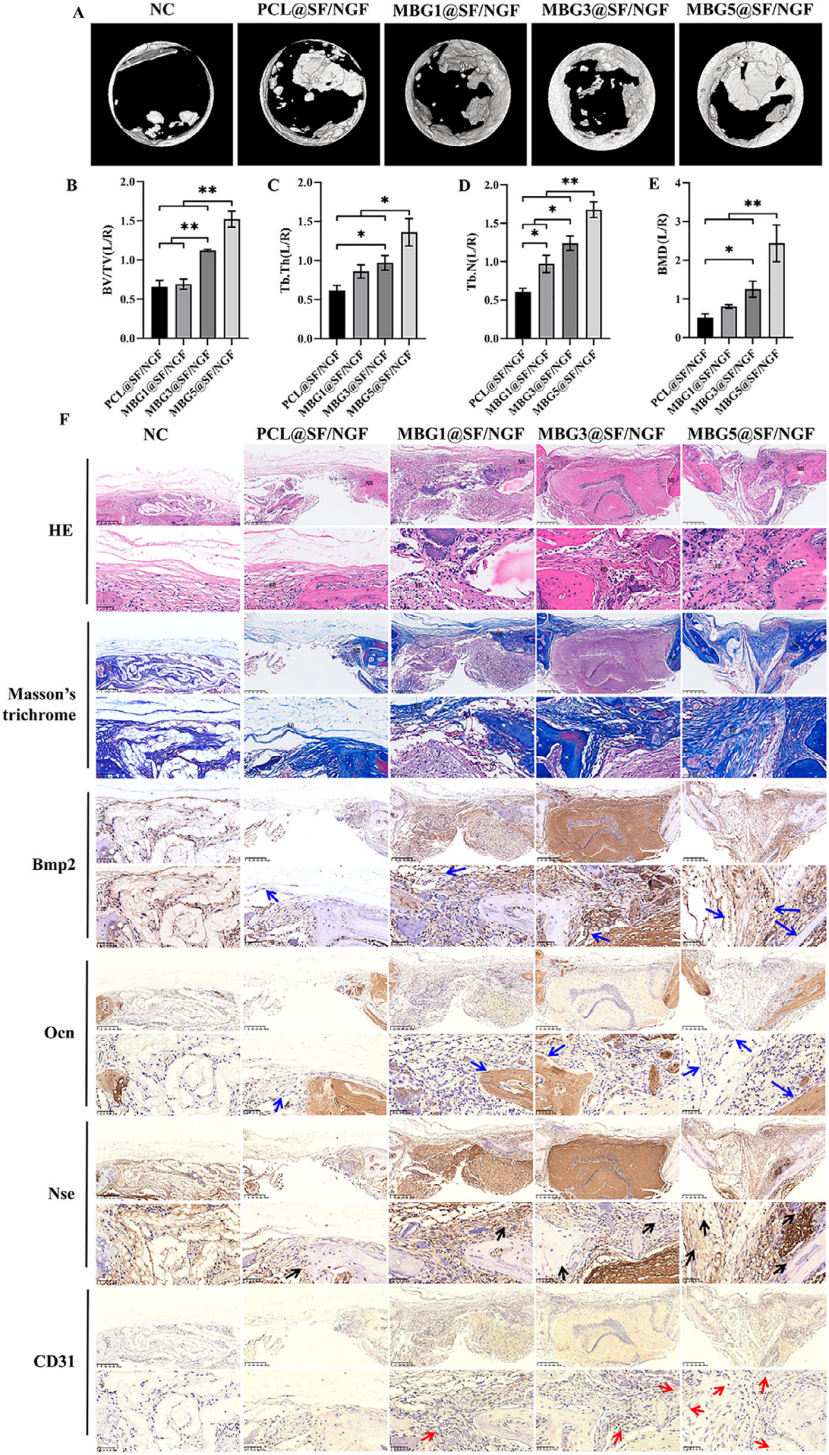
Bone repair efficacy of scaffolds in mice cranial defect model in vivo. (A) Three dimensional reconstructed micro CT images of the defect areas with these scaffolds transplanted for 4 weeks. (B) Quantitative analysis of new bone, including BV/TV (B), Tb.Th (C), Tb.N (D), and BMD (E). (F) Histological section measurement images. RB, Regenerative bone. NB, Native bone. The blue arrows indicate bone, the black arrow indicates nerves, and the red arrow indicates blood vessels.

To ultimately validate the bone repair efficacy of the ML‐optimized candidates, we used a mice calvarial critical‐size defect model to detect the osteogenic effect of these scaffolds in vivo. The result showed that the mice transplanted with MBG5@SF/NGF scaffold in defected area exhibited significantly superior new bone formation capacity compared to transplanted with other scaffold, and the area was largely covered with dense new formed bone tissue, achieving large part closure with good integration between the new bone and native bone, and the defected area transplanted with PCL@SF/NGF scaffold was exhibited with limited new bone volume failing to effectively bridge the defect, and the defected area transplanted with MBG1@SF/NGF scaffold was exhibited that evident bone regeneration, but the repair completeness was inferior to that transplanted with MBG5@SF/NGF scaffolds (Figure 9A). Furthermore, BV/TV (Figure 9B), Tb. Th (Figure 9C), Tb. N (Figure 9D) and BMD (Figure 9E) was significantly higher in MBG5@SF/NGF scaffold transplanted mice than in other scaffolds transplanted mice. These results suggested that the MBG5@SF/NGF scaffold has the potential ability in bone defect regeneration.

Moreover, to assess the morphology of the bone defect area, we used HE staining. The result showed that the defected area transplanted with the scaffold all formed bone marrow cavity, and abundant new bone tissue and regular trabecular structures lined with numerous osteoblasts in the MBG5@SF/NGF scaffold than in other scaffold filled defected area, indicating an active and healthy osteogenic environment. Besides that, Masson's trichrome staining showed that more Collagen was formed in the new bone regions of MBG5@SF/NGF scaffold transplanted area. Meanwhile, the expression locations of specific marker protein Bmp2, Ocn, Nse, and CD31 were detected through immunohistochemical analysis. Bmp2 is one of the most potent osteoinductive factors. Its core role is initiating and promoting osteogenic differentiation [18, 19]. Ocn, a non collagenous protein synthesized and secreted by mature osteoblasts, is a critical marker for bone matrix mineralization maturity, playing a key role in hydroxyapatite crystal deposition and calcium ion binding [20, 21]. Nse is a classic marker for neurons and neuroendocrine cells. Its elevation may indicate the activation of neuromodulatory mechanisms, which are part of the bone repair microenvironment. Bone tissue is rich in sensory nerve fibers. During injury and repair, these nerve endings release neuropeptides (e.g., CGRP, Substance P) known to directly promote angiogenesis, modulate inflammation, and stimulate osteoblast activity [22, 23, 24]. CD31 is a marker for vascular endothelial cells, and for the process of neovascularization within the tissue [25, 26]. The result demonstrated that Bmp2 and Ocn were expressed highly in MBG5@SF/NGF scaffold transplanted area in new bone formation, and Nse expression was also highly in Haversian canal in MBG5@SF/NGF scaffold transplanted area, and CD31 was also expressed highly in MBG5@SF/NGF scaffold transplanted area (Figure 9F). Taken together, these results indicated that higher Bmp2 expression delivers powerful osteogenic instructions, Ocn indicates progressing matrix mineralization maturity, increased Nse promotes nerve repair and also facilitates the process of bone repair, and elevated CD31 proved the generation of blood vessels. Therefore, MBG5@SF/NGF scaffold implantation effectively induced the reconstruction of a complete, physiologically regulated bone regeneration microenvironment to repair bone defect.

## 3 Discussion

Ideal bone repair materials should not only provide structural support and osteogenic induction activity but also possess the ability to regulate the local regenerative microenvironment, encompassing multiple biological processes such as neurogenesis, angiogenesis [27, 28]. Traditional biomaterial development heavily relies on empirical trial‐and‐error approaches, making it challenging to achieve rational design and performance optimization within complex multicomponent spaces. We report a ML‐accelerated screening strategy to identify promising compositional trends for osteogenic multicomponent MBG, followed by experimental validation in true MBG systems. We emphasize that ML serves as a screening tool to narrow down the vast composition space. This study successfully screened optimized formulations (MBG5) with superior osteogenic properties from a multicomponent MBG formulation space.

For complex formulation spaces involving multiple components such as Ca, Mg, Na, K, and Sr, traditional experimental screening suffers from low efficiency and struggles to reveal synergistic effects among components. This study integrated PCA (Figure 1A,D), t‐SNE (Figure 1B), response surface modeling, and nonlinear dimensionality reduction (Figure 1C,F) to visualize the high relative ALP activity region within the formulation space. This region correlates strongly with specific component ratios (e.g., Ca content), indicating significant synergistic or antagonistic interactions among constituents. Among various algorithms, the SVM model demonstrated optimal predictive performance (Figure 1J–N), fully leveraging its advantages in handling small sample, high dimensional nonlinear data. This is likely because SVM maximizes the margin between classes, making it less prone to overfitting than more complex ensemble methods when training data are limited. The standardized workflow employed in this study spanning data augmentation, feature engineering, model optimization, and validation enabled efficient prediction from 169 initial formulations to tens of thousands of virtual formulations (Figure 3). This approach successfully identified 126 high‐potential candidate formulations (1.26%), significantly accelerating the screening process while providing an interpretable, generalizable computational design framework. In the meantime, we acknowledge that our low‐performance control (MBG1) was selected from within the ML‐predicted high‐potential pool based on its relatively lower mineralization, rather than from the predicted low‐potential pool. This design choice, while intentionally made to test the effectiveness of experimental fine‐screening within a narrow activity range, nonetheless represents a less stringent negative control than selecting the absolute lowest performer across all formulations. The fact that MBG5 still exhibited significantly superior performance over MBG1 across all osteogenic and neuro‐inductive assays underscores the robustness of our screening cascade. These correlative observations are suggestive of potential functional interactions among the ionic components, but they do not establish causality. We therefore interpret these findings as hypotheses that require systematic experimental testing such as single element omission or substitution experiments to definitively confirm synergistic effects. Here follows our hypotheses. First, Ca‐based materials dominate. Ca is a key constituent of bone mineral, and predominates in all high‐performance formulations, mirroring mineral proportions in natural bone. In the second round screening tests of 126 formulations, we found that osteogenic performance decreased when the Ca content fell below 80% (formulations 43, 44, 54). Second, synergistic involvement of key functional elements is essential. We hypothesize that Sr and Mg may function through complementary pathways to exert synergistic effects. Sr is known to activate CaSR and Wnt signaling pathways, promoting osteoblast differentiation while inhibiting osteoclast activity [29], whereas Mg serves as an essential cofactor for multiple enzymes, playing a critical role in cell adhesion, proliferation, and early ALP activity [30]. The concurrent presence of these two ions at specific ratios in our high‐performance formulations suggests a potential complementarity that warrants systematic factorial validation. Calcium citrate ratios are generally higher in high osteogenic performance formulations than in low osteogenic performance groups. It is important to emphasize that citrate was not an input variable in our ML model. The introduction of citrate was an empirical, literature‐driven design choice rather than a model‐predicted outcome. The final metal cation ratios (Ca:Mg:Na:K:Sr) in all MBG formulations remained strictly consistent with the ML‐screened values. Citrate should therefore be viewed as an additional functional adjunct, like analogous to NGF in the shell that may contribute to the overall bioactivity of the scaffold, but whose effects were not part of the ML prediction. The potential role of citrate in regulating Ca^2+^ release kinetics and providing metabolic energy via the TCA cycle is proposed as a hypothesis for future investigation. We tentatively propose that citrate ions may possess synergistic regulatory potential that they could modulate Ca^2+^ release kinetics through chelation and provide metabolic energy via entry into the tricarboxylic acid cycle. However, this hypothesis requires dedicated experimental validation in future studies. Na and K may play a Balance Regulatory role. Na and K are indispensable for maintaining cell membrane potential and intracellular osmotic pressure equilibrium, forming the foundation for normal cellular physiological activity. In the optimal ratios identified through model screening, the proportions of these two ions are maintained within a relatively stable and moderate range, avoiding potential cytotoxicity or functional inhibition caused by excessively high or low levels.

The optimal formulation MBG5 in MBG5@SF/NGF scaffold demonstrated comprehensive biological properties that surpassed those of single osteogenic functions. Both formulation scaffolds significantly promoted BMSCs viability, ALP activity expression, and late‐stage mineralized nodule formation in vitro (Figure 5A–C), while upregulating osteogenic‐specific genes (*Runx2*, *Opn*, *Ocn*, etc.) and proteins (Runx2) (Figure 6). Additionally, the materials exhibited favorable antimicrobial activity (Figure 5G) and neuron affinity. These scaffolds manifested as promoting neural stem cell proliferation, upregulating the expression levels of neural specific genes (*Map*, *Nse*) (Figure 7), and Nse protein expression level was also increased in bone defect areas in vivo (Figure 9F). These results concluded that MBG5@SF/NGF scaffolds are not only passive scaffolds but also active platforms capable of actively remodeling the neuro‐bone coupled regenerative microenvironment. Studies have demonstrated the regulatory mechanism of the brain for bone healing in traumatic brain injury, injured neurons release exosomes enriched with pro‐osteogenic miRNAs that target osteoprogenitor cells, thereby promoting bone formation [31, 32, 33]. We hypothesize that since both skeletal and neural systems develop early in embryogenesis, brain injury reactivates these early developmental mechanisms. This reactivation triggers osteogenesis while simultaneously promoting the release of exosomes and related factors from neurons. The active growth of the nervous system stimulates bone formation, thereby creating a synergistic relationship where neural regeneration promotes bone development, which is supported with our results. Additionally, we postulate that there is a synergistic interplay among MBG, NGF, and SF in promoting osteogenesis. The osteogenic activity of the MBG@SF/NGF scaffold arises not merely from the sum of individual contributions from MBG, NGF, and SF, but rather from a mutually reinforcing positive feedback loop between osteogenesis and neurogenesis. On one hand, MBG releases bioactive ions that directly promote BMSC osteogenic differentiation via the Erk1/2‐Runx2 pathway (Figure 6), while simultaneously creating an ion microenvironment favorable for neural differentiation. On the other hand, NGF released from the SF shell drives neural lineage differentiation (Figure 7), and the resulting neural cells and progenitors secrete neuropeptides such as CGRP, substance P, and neurotrophins that further enhance osteoblast activity through paracrine signaling [34]. CGRP has been shown to activate Runx2 via the cAMP/PKA pathway, and substance P stimulates MAPK/Erk signaling in osteoblasts [35]. This steogenesis to neurogenesis to osteogenesis positive feedback loop is amplified by the core–shell architecture, which enables sustained NGF release while maintaining MBG‐mediated ion supply. We hypothesize that this synergistic loop, rather than any single component alone, underpins the superior regenerative performance of MBG5@SF/NGF in vivo (Figure 9).

At the signaling pathway level, the MBG5@SF/NGF scaffold is associated with elevated p‐Erk1/2 phosphorylation and subsequent upregulation of Runx2 at both mRNA and protein levels (Figure 6), along with its nuclear translocation. We propose that the p‐Erk1/2‐Runx2 axis represents a plausible candidate pathway through which the scaffold may exert the osteogenic effects based on that Runx2 is a master transcription factor for osteogenesis. While we observed consistent associations between MBG5@SF/NGF treatment, p‐Erk1/2 phosphorylation, Runx2 expression, and osteogenic differentiation, these data do not establish a causal signaling cascade. To unequivocally demonstrate that the scaffold promotes osteogenesis via the p‐Erk1/2‐Runx2 pathway, future studies should be emploied such as Pharmacological inhibition and Genetic approaches.

In a mouse skull critical‐sized defect model, the MBG5@SF/NGF scaffold induced substantial highly mature new bone formation within 4 weeks. The BV/TV and Tb. Th significantly performed better than the other scaffolds (Figure 9), with bone growth occurring alongside scaffold degradation. Histological and immunohistochemical findings provided deeper insights. Masson's trichrome staining revealed abundant blue Collagen deposition, and positive expression of Bmp2 and Ocn confirmed active osteogenic differentiation and matrix expression within the new bone suggested the ingrowth of new nerve fibers, and CD31‐positive signals indicated concomitant angiogenesis (Figure 9F). This aligns closely with the concept of neurogenic bone regeneration, where successful bone repair requires the restoration of innervation to regulate blood supply, sensory perception, and function. Furthermore, osteogenesis is not merely about bone formation but also about achieving physiological function. Research showed that necessitate vascular and neural development, meaning the rate of bone formation cannot be excessively rapid, and it must allow space for nerve and blood vessel growth, which in turn further promotes osteogenesis formed a positive feedback loop [36, 37, 38]. In our study, the shell of the scaffold was loaded with NGF, while the core of the scaffold consisted of MBG. When the scaffold was implanted, the shell releasing NGF to promote neurogenesis in the beginning, followed by the release of osteogenic functional elements from the core, which induced bone regeneration. However, the 4 week observation period captures only early‐stage bone healing and does not address scaffold degradation kinetics, long‐term remodeling, or functional biomechanical properties. The degradation behavior and structural integrity of the core–shell scaffold over time remain to be characterized, as do the sustainability of bone regeneration beyond 4 weeks and the mechanical competence of newly formed bone. While Nse and CD31 staining suggest neural and vascular ingrowth, the functional connectivity of these networks requires further validation. We will conduct further research, such as extended time points, degradation profiling, and biomechanical testing. For another, we observed the presence of inflammatory cell infiltration in the H&E sections at 4 weeks post‐implantation (Figure 9F). This observation is consistent with the normal foreign body response (FBR) that inevitably accompanies any biomaterial implantation. The FBR involves an acute inflammatory phase followed by monocyte or macrophage recruitment, which is a physiological cascade necessary for tissue repair rather than a pathological event. Notably, the MBG5@SF/NGF group concurrently exhibited robust new bone formation, Haversian canal‐like structures, and CD31‐positive neovascularization (Figure 9F), suggesting that the inflammatory response was in a resolving phase rather than progressing toward chronic inflammation. This resolving pattern is consistent with the known immunomodulatory properties of Sr^2+^ and Mg^2+^, which have been reported to promote macrophage polarization from the pro‐inflammatory M1 phenotype toward the pro‐regenerative M2 phenotype [39]. Immunohistochemical staining will be employed in future studies with M1‐specific markers and M2‐specific markers essential to systematically characterize the immunomodulatory capacity of the MBG@SF/NGF scaffold. This study marks this critical phenomenon in ML‐designed MBG scaffold, demonstrating the ability of the scaffold to effectively induce the regeneration of physiologically functional, neurovascularized bone tissue.

## 4 Conclusion

In this study, we construct a ML‐driven screening strategy to design multicomponent MBG‐based scaffolds and demonstrated their osteogenic, and neurogenic functions in vitro and in vivo. We discovered that the optimized MBG5@SF/NGF scaffold promotes osteogenic differentiation of BMSCs in conjunction with p‐Erk1/2 activated Runx2 signaling pathway. Notably, the scaffold also enhances neuroinductive properties, as evidenced by elevated expression of neural‐specific markers and neuron‐like morphology. In addition, in a critical‐sized cranial mice defect model, MBG5@SF/NGF scaffolds robustly stimulated new bone formation accompanied by neural ingrowth and vascularization. Taken together, this study not only presents a ML‐accelerated framework for designing complex biomaterials but also proposes MBG5@SF/NGF as the most promising multifunctional scaffold for neurovascularized bone regeneration.

## 5 Experimental Section

### 5.1 Cell Cultivation

Mouse bone marrow mesenchymal stem cells (BMSCs, Abiowell, China) were cultured with Dulbecco's Modified Eagle Medium (DMEM, Gibco Life Sciences, USA) supplemented with 10% fetal bovine serum (Zhejiang Tianhang Biotechnology, China), 1% penicillin‐streptomycin (Gibco Life Sciences, USA). The mineralization medium was the medium added with 50 µg/mL ascorbic acid (Beyotime Biotechnology, China), 10 mm β‐glycerophosphate (BBI Life Sciences Corporation, China), and 100 nm dexamethasone (Beyotime Biotechnology, China). Additionally, to induce neural differentiation in BMSCs, cells were first cultured in neurobasal medium (Gibco, USA) added with B27 (1X, Gibco, USA), 20 ng/mL fibroblast growth factor (FGF, Pepro Tech, USA), and 20 ng/mL epidermal growth factor (EGF, Pepro Tech, USA) for 7 day. Next, the medium was switched to Neurobasal medium containing B27 (1X), and cells were cultured for another 7 days.

### 5.2 High Throughput Experiment and Dataset Construction

Calcium (Ca, part from calcium nitrate, the other part from calcium citrate), magnesium (Mg, from magnesium nitrate), sodium (Na, from sodium nitrate), potassium (K, Frompotassium nitrate), and strontium (Sr, from strontium nitrate) were selected as the multicomponent for the bone mimetic matrix material. Under the constraint of constant total cation molar quantity across five element components, 169 distinct molar percentage ratios were generated. Using a fully automated high‐throughput pipetting workstation (ZUOFEI, China), each component was completely dissolved in 5% hydrochloric acid. Solutions were then mixed according to the designed ratios and transferred into 96‐well plates. Subsequently, the plates were placed in an oven and dried at 37°C for 24 h to form the component mixed materials. BMSCs were seeded on these materials at a density of 5 × 10^3^ cells per well into the 96‐well plates. Cell viability was performed using the Cell Counting Kit‐8 (CCK‐8, Dojindo, Japan) assay. 10 µL CCK‐8 solution was added to each well for incubating at 37°C for 4 h, and was measured under the absorbance at 450 nm (A_450_). Cell differentiation was detected through alkaline phosphatase (ALP) activity. 6 mm *p*‐nitrophenol inorganic phosphate (PNPP) solution was incubated with cells at 37°C for 30 min, and was measured under absorbance at 405 nm (A_405_). To comprehensively evaluate the osteogenic induction efficiency of materials, relative ALP activity was calculated for each group = (ALP value) / (CCK‐8 value) = A_405_/ A_450_. To construct the binary classification labels, a threshold of 0.83 was empirically selected based on the following considerations. On the one hand, relative ALP activity ≥ 0.83 corresponds to a substantial elevation above baseline levels over three times, representing a biologically meaningful enhancement of early osteogenic differentiation. On the other hand, in our dataset, this threshold yielded high osteogenic potential comprising <10% of the 169 samples, ensuring that the model was trained to identify the most exceptional performers among a large pool of candidates, which is a scenario that closely mimics real high‐throughput screening challenges. As a result, samples with value ≥0.83 are regarded as High Osteogenic Potential (Label 1), while the remainder are regarded as Low Osteogenic Potential (Label 0), thereby constructing a binary classification dataset.

### 5.3 ML Modeling and Prediction

169 datasets were randomly split into training and testing sets at a 7:3 ratio using stratified sampling to maintain category proportions. Z‐score normalization was performed on the multicomponent features (mole percentage). The calculation formula of Z‐score is shown in Equation (1), X represents the original data value, μ represents the mean of the data, σ represents the standard deviation of the data. SMOTE oversampling algorithm (k‐nearest neighbors = 10) was applied within the cross‐validation loop on the training set while keeping the testing set unchanged to prevent data leakage to address class imbalance in the training set. The principle of SMOTE is shown in Equation (2). X_new_ represents the generated sample, X_min_ represents the original minority sample, X represents sample randomly selected from the neighboring samples, λ represents a random number form 0 to 1. Then, eight classical ML algorithms were trained on the training set, Logistic Regression, SVM (kernel: radial basis), Decision Tree, Random Forest, Gradient Boosting, K‐Nearest Neighbors (KNN), Naive Bayes, and Multi‐Layer Perceptron (MLP) in the Scikit‐learn library. The decision function of SVM is shown in Equation (3), and the radial basis function is shown in Equation (4). In Equation (3), f(x) represents the predicted output (e. g., 0 or 1), sign represents the symbolic functions, n represents the number of samples, α represents the lagrangian multiplier corresponding to the sample, y represents the true labels of the training samples, K represents the kernel function, b represents the offset (intercept). In Equation (4), X_i_ and X_j_ represent the inner product of two samples in a high‐dimensional feature space, the exp represents the exponential function, γ represents a parameters, ‖*X_i_* − *X_i_* ‖^2^ represents the Euclidean distance.

(1) $$Z=\frac{X\;-\;\mu }{\sigma }$$

(2) $$X_{\mathrm{new}}=X_{\min}+\lambda \left(X-\;X_{\min}\right)$$

(3) $$f\left(x\right)=Sign(\sum_{i=0}^{n}\alpha_{i}y_{i}K\left(x_{i},x\right)+b)$$

(4) $$K\left(X_{i},X_{i}\right)=\exp\left(-\gamma {\left\|X_{i}-X_{i}\right\|}^{2}\right)$$

Model performance was evaluated using fivefold cross‐validation. Primary metrics included accuracy, precision, recall, and F1 score. The model with the best overall performance on the testing set was selected as the final prediction model. The measurement formulas are shown in Equations ((5), (6), (7), (8)). In binary classification, True Positive (TP), True Negative (TN), False Positive (FP), and False Negative (FN) are the four outcomes from a confusion matrix. TP and TN are correct predictions, while FP and FN are two types of wrong predictions. Uniformly generate 10 000 candidates across the entire space under the constraint that the total molar percentage of all six components sums to 100%. Screening out all candidates classified as high osteogenic potential (Label 1) through applying the trained optimal model to predict these candidates.

(5) $$Accuracy=\frac{TP+TN}{TP+TN+FP+FN}$$

(6) $$Precision=\frac{TP}{TP+FP}$$

(7) $$Recall=\frac{TP}{TP+FN}$$

(8) $$F1-score=\frac{2\times precision\times recall}{precision+recall}$$

### 5.4 Secondary Screening Based on Mineralization Capacity

All candidates with high osteogenic potential identified through the initial ML screening were screened a second times. BMSCs were seeded on these materials at a concentration of 2.5 × 10^4^ cells/mL into six well plates, cultured in medium for three days, and then the medium was switched to mineralization medium for the following 11 days. Alizarin red solution (40 mm, pH 4.2) was used to stain the calcium deposits. Bound dye was dissolved using 10% hexadecylpyridinium chloride solution (BBI Life Sciences Corporation, China) for quantitative comparison, and the absorbance was measured under 595 nm. Based on quantitative results, the four candidates with the highest mineralized nodule deposition (candidates 2–5) were selected as the high‐performance experimental candidate. Concurrently, one candidate with weaker mineralization capacity was randomly selected from those predicted as low osteogenic potential in the model as the low‐performance control group (candidate 1).

### 5.5 Preparation of Bioactive Fiber Scaffold

Calcium nitrate tetrahydrate, magnesium nitrate hexahydrate, sodium nitrate, potassium nitrate, strontium nitrate (Sigma–Aldrich, USA), and calcium citrate tetrahydrate (Sangon Biotech, China) were used. The total molar amount of cations was controlled as 0.01588 mol. Based on the selected ratios (candidate 1–5), the molar quantities of each cation within each candidate were calculated. Subsequently, the required masses of each nitrate corresponding to these molar quantities were determined. 4.8 g polyethylene oxide–polypropylene oxide–polyethylene oxide (P123) was dissolved in 300 mL sterile water with an additional 6 g 1,3,5‐trimethylbenzene (TMB), which is a hole expanding agent. Then, the calculated mass of each component according to the group ratios was added with the corresponding nitrates. 10 mL tetraethyl orthosilicate (TEOS), and 2.6 g of triethyl phosphate (TEP) was also dissolved in the solution. The pH was adjusted to 10.0 with ammonia solution. The product was obtained through settling at 90°C for 72 h, dried at 100°C for 10 h, and calcined in a muffle furnace at 550°C for 8 h. 0.1 g powder was put into 0.1 m 3‐Aminopropyltriethoxysilane (APTES) to get amino‐modified MBG.

The prepared MBG was named sequentially according to candidates 1–5 as MBG1‐5. Based on the selected formulations, a corresponding mass of calcium citrate was added to MBG, and both of them were grinded to mix completely. 2.5 mL electrospinning solution (polycaprolactone (PCL, 10%, w/v) / hexafluoroisopropanol (HFIP, Aladdin, China)), with 8.95 µg NGF (PeproTech Inc, USA) adsorbed into 6.25 mg SF as the shell solution (NGF/SF), and 2.5 mL electrospinning solution with 25 mg MBG as the core solution. The shell and core solutions were transferred into two syringes for coaxial electrospinning. The prepared composite fiber scaffolds were named sequentially according to candidate 1–5 as MBG1@SF/NGF, MBG2@SF/NGF, MBG3@SF/NGF, MBG4@SF/NGF, MBG5@SF/NGF, and coaxial fiber membranes containing only PCL in the core soluiton (PCL@SF/NGF) and uniaxially electrospinning PCL fiber scaffold (PCL) were regarded as control.

### 5.6 Ion Release Kinetics of MBG

To evaluate the ion release behavior of different MBG formulations, 10 mg of each MBG powder (MBG1‐MBG5) was dispersed in 30 mL of simulated body fluid (SBF) in centrifuge tubes. The suspensions were incubated in an orbital shaking incubator at 37°C with constant agitation at 100 rpm. At predetermined time points (day1, 3, 6, 9), 1 mL of the supernatant was collected and replaced with an equal volume of fresh SBF to maintain constant volume. The concentrations of released Ca^2+^, Mg^2+^, Na^+^, K^+^, and Sr^2+^ in the collected supernatants were determined using inductively coupled plasma optical emission spectrometry. All measurements were performed in triplicate, and cumulative ion release concentrations were calculated with correction for volume replacement.

### 5.7 Characterization of Bioactive Fiber Scaffold

In order to evaluate the morphology and composition of each candidate, we characterized these materials. First, Brunauer‐Emmett‐Teller (BET) experiments were conducted to verify whether the MBG have mesoporous structure. Alexa Fluor 594 and Fluorescein‐5‐isothiocyannate (FITC) were added to the shell and core electrospinning solutions for coaxial electrospinning, respectively. Fluorescence distribution was observed using Confocal Laser Scanning Microscope (CLSM, Leica TCS SP8, Germany) to confirm the core–shell structure. The morphology and distribution of the surfaces of these scaffolds were observed using scanning electron microscopy (SEM; JSM‐6700F, Japan). Simulated body fluid (SBF) solution was prepared by adding 0.225 g KCl, 0.230 g K_2_HPO_4_·3H_2_O, 0.311 g MgCl_2_·6H_2_O, 11.928 g HEPES, 0.293 g CaCl_2_, 0.072 g Na_2_SO_4_, 0.0008 mol NaOH, 5.403 g NaCl, 0.740 g NaHCO_3_, and 2.046 g Na_2_CO_3_ (all from BBI Life Sciences, China) in 1 L water for mineralization detection in vitro. These scaffolds were immersed in SBF and soaked at 37°C for 14 days, and were examined through SEM to observe the surface morphology of mineralization deposition. Characteristic functional groups of the scaffold were analyzed using Fourier Transform Infrared Spectroscopy (FTIR). Tensile testing of the fiber scaffolds (20 mm × 5 mm × 20 µm) was conducted using a dynamic mechanical testing machine (Bose ElectroForce, USA) to determine the elastic modulus. The calculation formula is shown in Equation (9). E represents the elastic modulus, σ represents the stress, ε represents strain, F represents the applied force, A represents the cross‐sectional area, ΔL represents the deformation of length, and L represents the original length.

(9) $$E=\frac{\sigma }{\varepsilon }=\frac{F/A}{\Delta L/L}\;$$

### 5.8 Antimicrobial Activity and Cell Migration of Bioactive Fiber Scaffold

Antimicrobial activity of these bioactive fiber scaffolds was detected through placing these scaffolds into the tubes and incubated with LB medium, then the medium were put onto the LB solid culture medium, and the bacterial suspension was also put onto it. The growth of the *E. coli* bacterial was observed. ImageJ was used to count the *E. coli* colonies number on each scaffold. Additionally, cell migration was determined through placing BMSCs in the upper chamber of a Transwell device, and the lower chamber was filled with culture medium. After incubating for 24 h, cells were fixed with 4% paraformaldehyde (Biosharp, China), and then were stained with crystal violet and were observed under a microscope to obtain images. ImageJ was used to conduct quantitative analysis of cell migration of each scaffold. Furthermore, cell adhesion was detected through BMSCs seeded on the scaffolds and cultured for 1 day. After washing, fixation, dehydration, and drying, SEM was used to observe cell morphology in scaffolds.

### 5.9 Western Blotting Analysis

Total proteins of BMSCs cultured on these scaffolds were extracted using Radio Immunoprecipitation Assay (RIPA) lysis buffer (Beyotime Biotechnology, China), and protein concentration was measured with the Bicinchoninic acid (BCA) assay kit (Thermo Fisher, USA). Proteins were separated by Sodium Dodecyl Sulfate Polyacrylamide Gel Electrophoresis (SDS‐PAGE), and then transferred to polyvinylidene fluoride (PVDF) membranes, and incubated with specific primary antibodies (p‐Erk1/2 (1:1000, Cell Signaling Technology, USA), Erk1/2 (1:2000, Beyotime Biotech, China), Runx2 (1:1000, Cell Signaling Technology, USA), Gapdh (1:2000, Beyotime Biotech, China), and Nse (1:1000, Beyotime Biotech, China)), and then incubated with corresponding HRP labeled secondary antibodies. Images were developed using Electrochemiluminescence (ECL, Pierce, USA) and quantified with ImageJ software.

### 5.10 Real‐Time Quantitative Polymerase Chain Reaction (RT‐qPCR) Analysis

Total RNA was extracted using TRIzol (Invitrogen, USA). Reverse transcription was performed using a reverse transcription kit (Invitrogen, USA). qPCR analysis was conducted using a SYBR Green PCR kit (Toyobo Co., Ltd., Japan) on a LightCycler 480 real‐time PCR system (Hoffmann‐La Roche, Switzerland). All these procedures were conducted according to the instruction manual. The following primers were used as the forward and reverse primers: *Osterix* (*Osx*) 5’ ‐GTCAAGAGTCTTAGCCAAACTC‐3’ and 5’ ‐AAATGATGTGAGGCCAGATGG‐3’; *Osteopontin* (*Opn*) 5’ ‐TCCAAAGCCAGCCTGGAAC‐3’ and 5’ ‐TGACCTCAGAAGATGAACTC‐3’; *Bone Morphogenetic Protein 2* (*Bmp2)* 5’ ‐CTGACCACCTGAACTCCAC‐3’ and 5’ ‐CATCTAGGTACAACATGGAG‐3’; *Runt‐related transcription factor 2* (*Runx2*) 5’ ‐GAATGCACTACCCAGCCAC‐3’ and 5’ ‐TGGCAGGTACGTGTGGTAG‐3’; *Type I Collagen* (*Col*) 5’ ‐CCTGGTAAAGATGGTGCC‐3’ and 5’ ‐CACCAGG TTCACCTTTCGCACC‐3’; *Osteocalcin* (*Ocn*) 5’ ‐CAATAAGGTAGTGAACAGAC‐3’ and 5’ ‐CTTCAAGCCATACTGGTCT‐3’; *Microtubule‐associated protein 2* (*Map2*) 5’ ‐ATCAGGAGACAGGGAGGAGAACT‐3’ and 5’ ‐AGGGGTAGTAGGTGTGGAGGTG‐3’; *Neuron‐specific enolase* (*Nse*) 5’ ‐ACCTGATGTTGGAACTGGATG‐3’ and 5’ ‐GCTGAGCAATGTGGCGATAG‐3’; *Glyceraldehyde 3‐phosphate de‐hydrogenase* (*Gapdh*) 5’ ‐CATGGCCTTCCGTGTTCCTA‐3’ and 5’ ‐CCTGCTTCACCACCTTCTTGAT‐3’. *Gapdh* served as the internal control, and relative expression levels were calculated using the 2^−ΔΔCt^) equation.

### 5.11 Immunofluorescence Staining

After BMSCs or BMSCs induced neural differentiation cells cultured on these scaffolds for 7 days, the cells were washed, fixed, and blocked with 10% horse serum. Then, the cells were incubated with primary antibodies (Runx2(1:100), Bmp2 (1:200), Actin (1:400), and Nse (1:100) (all purchased from Beyotime Biotech, China), followed by incubation with corresponding fluorescent secondary antibodies. The nuclear was stained with 4’,6‐Diamidino‐2‐phenylindole (DAPI, Beyotime Biotech, China). Images were captured through CLSM.

### 5.12 Transcriptome Sequencing

BMSCs were cultured on these scaffolds for 7 days, respectively. RNA was extracted and sequenced using an RNA sequencing (RNA‐Seq) platform (Novaseq 6000, Illumina, USA). Gene expression levels were analyzed through bioinformatics analysis.

### 5.13 Animal Model and Micro Computed Tomography (Micro CT) Analysis

Two symmetrical circular defects with a diameter of 2.8 mm (critical sized cranial defects) were created in the skull of 6‐week‐old male C57/BL6 mice. The scaffolds (PCL@SF/NGF, MBG1@SF/NGF, MBG3@SF/NGF, MBG5@SF/NGF) were implant on the left side, leaving the right side unimplanted as a negative control (NC), then the wounds were sutured. A 12‐h light‐dark cycle was maintained to preserve the circadian rhythm of mice. Skull samples were harvested 4 weeks post‐surgery. Scanning was performed using a Micro CT scanner (SkyScan 1276, Bruker, Belgium). Three‐dimensional reconstruction was conducted with the accompanying software, followed by quantitative analysis of the defect area for bone volume/tissue volume (BV/TV), trabecular thickness (Tb.Th), trabecular number (Tb.N), and bone mineral density (BMD).

### 5.14 Histological Analysis

The skulls were fixed in 4% paraformaldehyde and then decalcified in Ethylenediaminetetraacetic Acid (EDTA) for one month. Paraffin‐embedded sections (5 µm thick) were underwent Hematoxylin Eosin (HE) and Masson's trichrome staining. Sections were labeled with Bmp2 (1:100), Ocn (1:1800), Nse (1:300), and CD31 (1:600) antibodies (all from Affinity, USA) for immunohistochemical staining. All the images were observed using an optical microscope (NanoZoomer S360, HAMAMATSU, Japan).

### 5.15 Statistical Analysis

All these experiments were independently replicated at least three times. Data are expressed as mean ± standard deviation. Statistical analysis was performed using GraphPad Prism software. Comparisons among multiple groups were conducted using one‐way ANOVA followed by post hoc tests. Comparisons between two groups were performed using Student's t‐test. *p* <0.05 was considered statistically significant. ^*^ *p* <0.05 and ^**^ *p* <0.01.

## Author Contributions

**Kunlu Lin**: Investigation, Methodology, Writing – original draft, Conceptualization. **Ying Yang**: Data curation, Formal analysis, Investigation, Methodology, Writing – original draft. **Chuyao Zhou**: Data curation, Methodology, Writing – original draft. **Hanyue Mao**: Data curation, Methodology. **Zhiquan Zhang**: Data curation. **Libangxi Liu**: Data curation. **Xiaoying Yu**: Data curation. **Haoming Liu**: Methodology. **Long Liu**: Methodology, Writing – review & editing, Funding acquisition. **Xiaoyan Wang**: Investigation, Methodology, Supervision, Writing – review & editing, Funding acquisition.

## Funding

This project received financial support from the National Natural Science Foundation of China (No. 32371423) and the Innovation Research Foundation of National University of Defense Technology.

## Ethics Statement

All animal‐related experimental procedures were based on the National Research Council's Guide for the Care and Use of Laboratory Animals and were approved by the Animal Ethics Committee of Hunan Provincial Laboratory Animal Center (HNSE2025(5)006).

## Conflicts of Interest

The authors declare no conflicts of interest.

## Supporting information




**Supporting File 1**: advs77084‐sup‐0001‐SuppMat.docx.

**Supporting File 2**: advs77084‐sup‐0002‐Data.zip.

## Data Availability

The data that support the findings of this study are available from the corresponding author upon reasonable request.
