# Supplementary material for: Machine Learning‐Driven Design of Multicomponent Bone Inorganic Matrix Mimicking Scaffolds for Osteogenesis Enhanced by Neurogenesis
Source: Adv Sci (Weinh). 2026 Aug 27:e77084. Online ahead of print. doi: 10.1002/advs.77084 (PMC13519502; doi:10.1002/advs.77084)
Supplement: Supplementary file 1 [file ADVS-9999-e77084-s001.docx]

**Figure S1**

Figure S1. Result of ion release. Absolute ion release profile of Ca^2+^ (A), Mg^2+^ (B), Na^+^ (C), K^+^(D)and Sr^2+^ (E) ions from MBG1-5 samples immersed in SBF measured by ICP.
